# Patient-derived organotypic tissue cultures as a platform to evaluate metabolic reprogramming in breast cancer patients

**DOI:** 10.1016/j.jbc.2025.108495

**Published:** 2025-04-08

**Authors:** Teresa W.-M. Fan, Jing Yan, Carlos Frederico L. Goncalves, Jahid M.M. Islam, Penghui Lin, Mohamed M.Y. Kaddah, Richard M. Higashi, Andrew N. Lane, Xiaoqin Wang, Caigang Zhu

**Affiliations:** 1 Center for Environmental and Systems Biochemistry (CESB), Department of Toxicology and Cancer Biology, and Markey Cancer Center, University of Kentucky, Lexington, Kentucky, USA; 2 F. Joseph Halcomb III, M.D. Department of Biomedical Engineering, University of Kentucky, Lexington, Kentucky, USA; 3 Pharmaceutical and Fermentation Industries Development Center, City of Scientific Research and Technological Applications, Alexandria, Egypt; 4 Department of Radiology, University of Kentucky, Lexington, Kentucky, USA

**Keywords:** patient-derived organotypic tissue cultures, breast cancer, tumor metabolic reprogramming, stable isotope-resolved metabolomics, reverse phase protein array, gluconeogenesis, nucleotide synthesis

## Abstract

Patient-derived organotypic tissue cultures (PD-OTC) are unique models for probing cancer metabolism and therapeutic responses. They retain patient tissue architectures/microenvironments that are difficult to recapitulate while affording comparison of cancer (CA) *versus* matched noncancer (NC) tissue responses to treatments. We have developed a long-term culturing method for fresh and cryopreserved PD-OTC of breast cancer patients bearing invasive ductal carcinoma. Five PD-OTC came from patients with treatment-naïve primary ER^+^/PR^+^/HER2^-^ tumors while one came from a patient with neoadjuvant therapy for locally metastatic ER^low^/PR^-^/HER2^-^ tumor. They all exhibited tissue outgrowth in 1 month with some CA OTC harboring isolatable organoids and fibroblasts. We interrogated reprogrammed metabolism in CA *versus* paired NC OTC with dual ^2^H_7_-glucose/^13^C_5_,^15^N_2_-Gln tracers coupled with stable isotope-resolved metabolomic analysis. We noted variable activation of glycolysis, cataplerotic/anaplerotic Krebs cycle including reductive carboxylation, the pentose phosphate pathway, riboneogenesis, gluconeogenesis, *de novo* and salvage synthesis of purine/pyrimidine nucleotides, and ADP-ribosylation in CA PD-OTC. Altered metabolic activities were in part accountable by expression changes in key enzymes measured by reverse phase protein array profiling. Notably, Gln-fueled gluconeogenesis products were preferentially diverted to support purine nucleotide synthesis. When blocking this novel process with an inhibitor of phosphoenolpyruvate carboxykinase (3-mercaptopicolinic acid), metastatic, ER^low^/PR^-^/HER2^-^ CA OTC displayed compromised cellularity, reduced outgrowth, and disrupted growth/survival-supporting metabolism but the matched NC OTC did not. Thus, our PD-OTC culturing method not only promoted understanding of actual patient’s tumor metabolism to uncover viable metabolic targets but also enabled target testing and elucidation of therapeutic efficacy.

As for other human cancers, metabolic reprogramming in breast cancer (BC) is a key contributing factor in its development, progression, and metastasis (1, 2, 3). Many aspects of central metabolism, including glycolysis, the pentose phosphate pathway (PPP), the Krebs cycle, one-carbon metabolism, glutaminolysis, anti-oxidation, and lipid metabolism have been shown to be dysregulated in BC cells or tissues (1, 2, 3). This knowledge not only advances understanding of BC biology but also helps identify novel therapeutic target(s) and markers for disease/drug response, early diagnosis, and prognosis (3, 4, 5, 6, 7, 8, 9). However, with a few exceptions (10, 11, 12, 13, 14), metabolic traits in BC are primarily derived from transcriptomic/protein analyses and/or proximal measurements such as oxygen consumption/extracellular acidification in BC cell lines (1, 2, 3). Gene expression alone can fail to accurately define metabolic networks as it can deviate from protein expression and does not account for posttranscriptional and posttranslational modifications (PTM) that alter enzyme activities. Even altered protein expression or PTMs do not completely define metabolic activity, due to the presence of allosteric regulation by metabolites, protein–protein interactions, or alternative (moonlighting) functions of metabolic proteins. For example, glycolytic aldolase A (ALDOA) exhibits aldolase-independent stimulation of protein translation in cancer cells while the key gluconeogenic enzyme phosphoenolpyruvate carboxykinase 1 (PCK1) promotes lipogenesis by phosphorylating INSIG1/2 (9). Moreover, proximal analyses lack the power to resolve interconnecting metabolic pathways. Stable isotope tracer-based approaches such as stable isotope-resolved metabolomics (SIRM) overcome these shortcomings by broadly and rigorously mapping metabolic network activities in cells, tissues, and even whole organisms including human subjects (14, 15, 16, 17, 18, 19). Using SIRM, we found that BC cells display enhanced pyruvate carboxylation and disparate metabolic activities between *in vitro* cultures and tumor xenografts, presumably reflecting the influence of the tumor microenvironment (TME) (14). Interestingly, pyruvate carboxylase (PC)-dependent anaplerosis was found to be enhanced in lung metastases of BC cells by adapting to the lung microenvironment (20). These studies highlight the importance of TME on metabolic reprogramming in BC, as the case for other solid cancers such as lung cancer (21).

*In vitro* 2D cell cultures cannot model the TME because they lack cell–cell and cell–matrix interactions. 3D spheroid models and *in vivo* tumor xenografts including patient-derived xenografts or syngeneic transplants can provide some forms of TME but cannot recapitulate the TME in human cancer patients. Patient-derived organoids and organotypic tissue cultures (PD-OTC) are more recent models that best represent the native TME of individual patients while affording full flexibility for culture manipulations and treatments without influences from other organs (22, 23, 24, 25). The PD-OTC model has the added advantage of maintaining the native tissue architecture and cellularity while enabling comparison of treatment responses between cancerous (CA) and matched noncancerous (NC) tissues (16, 17, 18, 26). PD-OTC models for human breast cancer (BC-PD-OTC) have been shown to maintain tissue morphology and cell viability for 7 days as *ex vivo* cultures while displaying variable sensitivity to drug treatments (27). We have recently established an improved protocol for *ex vivo* culturing of BC-PD-OTC and shown hyperactive glucose uptake and mitochondrial membrane polarization in CA *versus* NC OTC *via in situ* optical spectroscopic assays (28). However, to the best of our knowledge, no metabolic studies have been conducted on BC-PD-OTC to gain understanding of their metabolic networks.

Here we report a culturing method that sustained metabolic viability of freshly resected and cryopreserved CA and their matched NC BC-PD-OTC for greater than a month, during which CA tissue outgrowth occurred that harbored isolatable organoids and fibroblasts. We also utilized SIRM coupled with a dual tracer cocktail of [^2^H_7_]-glucose + [^13^C_5_,^15^N_2_]-glutamine (multiplex SIRM or mSIRM) to extensively define metabolic reprogramming *ex vivo* in CA *versus* NC OTC. Five of these OTC were derived from BC patients bearing invasive ductal carcinoma (IDC), which is positive for estrogen receptor (ER^+^) and progesterone receptor (PR^+^) but negative for human epidermal growth factor receptor 2 (HER2^-^). The sixth OTC was obtained from a patient with low ER (ER^low^), PR^-^, and HER2^-^ IDC. We found that CA OTC variably displayed activated glycolysis, cataplerotic, and anaplerotic Krebs cycle including reductive carboxylation, the pentose phosphate pathway, riboneogenesis (RNG), gluconeogenesis, *de novo* and salvage synthesis of purine/pyrimidine nucleotides, O-linked GlcNAcylation, and ADP-ribosylation, compared with matched NC OTC. A novel finding was the preferential diversion of Gln-fueled gluconeogenesis (GNG) products to support purine nucleotide synthesis. Blocking this process using a PCKi (3-mercaptopicolinic acid, 3-MPA) led to reduced outgrowth, tissue cellularity, and growth/survival-supporting metabolism in metastatic, ER^low^/PR^-^/HER2^-^ CA *versus* matched NC OTC.

## Results

### PD-BC-OTC show increased medium acidification and outgrowth of organoid/fibroblast-like structures

We applied the novel culturing method to six pairs of thinly sliced (750 μm) matched CA and NC breast tissues each resected from a BC patient and embedded in membrane inserts with Matrigel as described in Experimental procedures and illustrated in Fig. S1*A*. All BC tissues were classified as IDC, 5 of which (CZ016, CZ019-022) were treatment-naïve, ER^+^, PR^+^, and HER2^-^ while one (CZ017) was PR^-^/HER2^-^ with low expression of ER and underwent neoadjuvant therapy prior to surgery (Fig. S1*H*). The OTC of CZ019 were recovered from cryopreservation while the other OTC were from freshly resected patient tissues. Regardless of patient attributes or tissue handling, all OTC maintained structural integrity (*e.g.* Fig. S1*D*) and abundance of ER^+^ and Ki67^+^ (proliferative) cells (*e.g.* Fig. S1*E*) while the CA OTC displayed outgrowth of organoid- and fibroblast-like structures (*e.g.* Fig. S1*B*) during 1 month of culturing. We were able to isolate these Matrigel-embedded structures as organoids and fibroblasts from the CA OTC of CZ017 and CZ019 as illustrated in Fig. S1, *F* and *G*, respectively, despite the very slow growth of the isolated organoids.

Equally important, all OTC remained metabolically active as evidenced from the acidification of culture media. We thus employed a multiplex SIRM (mSIRM) approach to interrogate the metabolic network activity of paired CA *versus* NC OTC. This approach utilizes multiple stable isotope tracers in the same experiment to achieve greater metabolic pathway coverage than the single tracer approach, while enabling a direct comparison for the utilization of multiple fuel sources without any sample batch effects (17, 29, 30). Another important advantage of mSIRM is the much reduced demand for human patient tissues, which are always limited in supply. Further, the use of tracers is much more robust and suited for defining and uncovering novel aspects of metabolic networks than metabolite profiling alone. The matched CA and NC OTC design enabled a comprehensive understanding of reprogrammed cancer metabolism in human BC patients and its dependence on individual patients’ attributes without interferences from variance in physiology, nutrition, and/or genetics.

### mSIRM analysis reveals reprogrammed central metabolism in human BC OTC

We used a cocktail of ^2^H_7_ (D_7_)-glucose + ^13^C_5_,^15^N_2_-glutamine in all six tracing experiments to track the metabolic transformations of these two key fuels simultaneously. The pathways traced included glycolysis, the Krebs cycle, glutathione (GSH) biosynthesis, the PPP, GNG, and pyrimidine/purine nucleotide biosynthesis.

### Activation of glycolysis/GSH synthesis/anaplerosis of the Krebs cycle and disruption of the Krebs cycle in the CA OTC of BC patients

**Figure 1:**
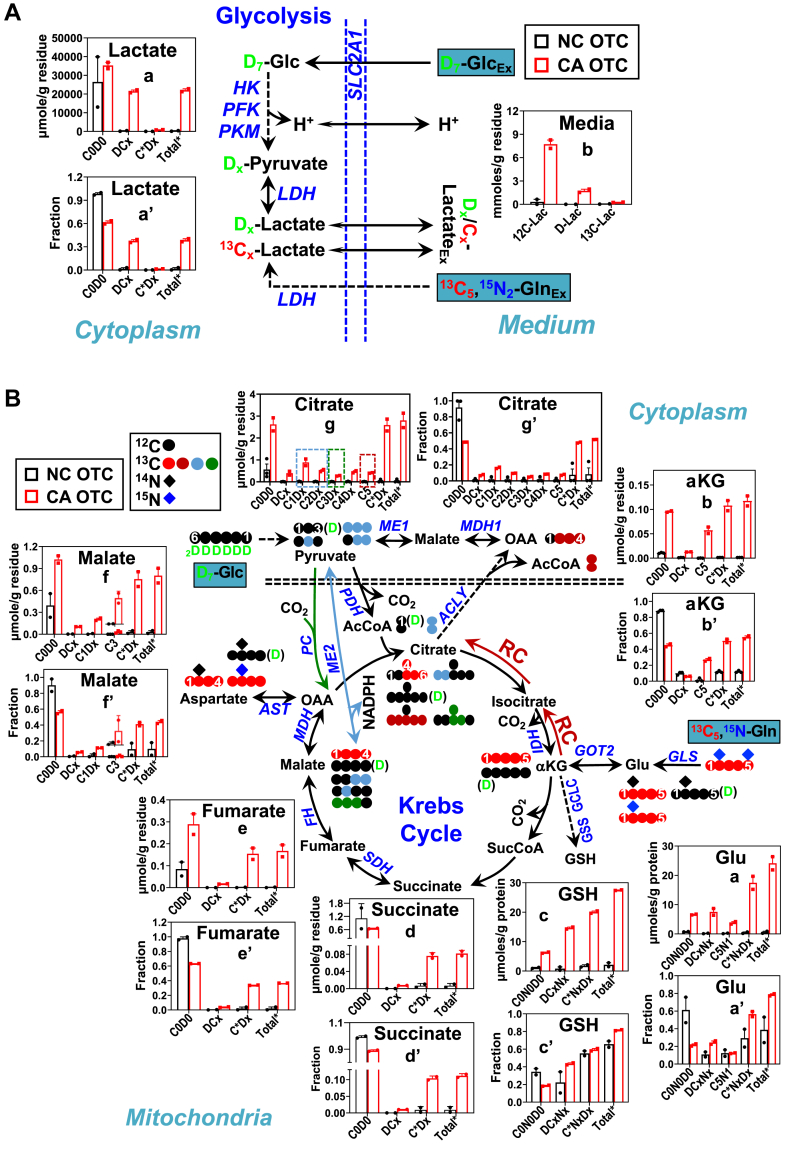
**CA PD-OTC of CZ019 displays enhanced glycolysis and noncanonical Krebs cycle metabolism.** Cryopreserved CA and NC OTC of CZ019 patient (n = 2 biological replicates) were cultured in D_7_-glucose + ^13^C_5_,^15^N_2_-Gln-containing BCOM for 2 days before harvest for SIRM analysis by IC-UHR-FTMS (in μmole/g residue and fraction) except for the analysis of medium lactate (in μmole/g residue; panel Ac) by ^1^H NMR. The schemes in *A* and *B* tracks D, ^13^C (,,,), ^15^N (), ^12^C (), and ^14^N () atoms in glycolysis, the Krebs cycle, glutaminolysis, and glutathione (GSH) synthesis. , ,  in (*B*) denote reductive carboxylation (RC), cataplerotic pyruvate carboxylase (PC), malic enzyme (ME) pathways, respectively. C0D0/C0N0D0: all ^12^C, ^14^N, ^1^H or unlabeled; DCx/DCxNx: all D labeled with variable (0-x) number of ^13^C and/or ^15^N; N∗CxDx: all ^15^N labeled with 0-x number of ^13^C and/or D; C1-4Dx: ^13^C_1_ to ^13^C_4_ with 0-x number of D; C∗Dx/C∗NxDx: all ^13^C labeled with 0-x number of D and/or ^15^N; C3: ^13^C_3_, C5: ^13^C_5_, C5N1: ^13^C_5_,^15^N_1_; Total∗: all labeled; (D): Unspecified D labeled position and number. In (*A*), enhanced glycolysis in CA () *versus* NC () OTC was evident by increased buildup of tissue D-labeled pyruvate/lactate (DCx) and increased release of D-lactate (D-Lac) into the medium. Also shown for CA *versus* NC OTC was increased release of unlabeled lactate (^12^C-Lac). *B*, shows increased D, ^13^C, and ^15^N labeling (in μmole/g residue and fraction) of glutaminolytic products (Glu, α-Ketoglutarate or αKG), Krebs cycle metabolites, and GSH in CA *versus* NC OTC. See Table S3 for statistics. AcCoA, SucCoA, acetyl and succinyl coenzyme A; ACLY, ATP citrate lyase; AST, aspartate aminotransferase or SLC17A5; CS, citrate synthase; FH, fumarate hydratase; GCLC, Glutamate-cysteine ligase catalytic subunit; Glc, glucose; GLS, glutaminase 1; GSS, glutathione synthetase; HK, hexokinase;LDH, PDH, IDH, OGDH, SDH, and MDH, lactate, pyruvate, isocitrate, oxoglutarate, succinate, malate dehydrogenase; OAA, oxaloacetate; PFK, phosphofructokinase; PKM, pyruvate kinase M; SLC2A1, glucose transporter type 1.

Lactate release into the culture medium accounts for most of the glucose metabolism *via* glycolysis, as shown for CZ019 OTC in Figure 1*A*. We saw greatly increased release of extracellular D-labeled lactate (D-Lac, b) for CA *versus* NC OTC, which signifies much enhanced oxidation of D_7_-glucose *via* glycolysis. This is consistent with elevated levels and fractional enrichment of D-labeled tissue lactate (DCx, a and a’). We also saw even greater increases in the release of unlabeled lactate (^12^C-Lac, b) into the medium by CA *versus* NC OTC, which suggests enhanced metabolism of unlabeled precursor(s) such as glycogen. A similar increase in D-labeled and unlabeled lactate release was evident in the CA *versus* NC OTC of all 5 other patients (Figs. S2*A*a, j and S3–S5*A*a). However, the amount of Gln-derived lactate (^13^C-Lac) in the medium was highly variable, that is, it was high and significantly elevated for the CA *versus* NC OTC of CZ016 and CZ017 (Figs. S3 and S4*A*a) but was negligible for the CZ019 to 022 counterparts (Figs. 1*A*b, S2a/j, and S5*A*a). This suggests highly variable lactate synthesis from Gln among different BC patients’ OTC. Thus, lactate dehydrogenase blockade would be the best strategy for glycolysis-targeted BC therapy to block all sources of lactate production.

In addition to glycolysis, glucose and Gln can be transformed in the Krebs cycle *via* the cataplerotic and anaplerotic routes initiated by the pyruvate dehydrogenase (PDH) and PC/malic enzyme (ME) reactions, respectively. Figure 1*B* shows the transformation pathways of the two fuels in the Krebs cycle along with the expected D, ^13^C, and ^15^N labeling patterns of the resulting products. In particular, the ^13^C labeling patterns of citrate isotopologs informs on the activity of Krebs cycle reactions plus glutaminolysis. ^13^C_5_,^15^N_1_ (C5N1)-Glu, and ^13^C_5_ (C5)-α−ketoglutarate (αKG) results from glutaminolysis () while ^13^C_4_-bearing (C4Dx)-citrate is derived from glutaminolysis plus the canonical Krebs cycle. ^13^C_5_ (C5)-citrate is most likely a product of reductive carboxylation (RC, ) *via* reversing isocitrate dehydrogenase action as glutaminolysis + canonical Krebs cycle should produce ^13^C_5_,D-bearing citrate, which was present at much lower levels (data not shown). ^13^C_3_-bearing (C3Dx) citrate can result from malic enzyme 1 (ME1 )–catalyzed malate to pyruvate plus the pyruvate carboxylase (PC ) reaction while ^13^C_2_-bearing (C2Dx) citrate may reflect ME1 plus ME2 activities. ^13^C_1_-bearing (C1Dx) citrate is generated from ^13^C_2_-pyruvate *via* the reverse ME2 reaction. Moreover, the sum of all ^15^N-labeled (N∗CxDx) isotopologs of Asp (*cf.* Figure 3*A*) results from glutaminolysis plus transamination reactions while all ^13^C- (C∗Dx) and D-bearing (DCx) species (Total∗) for the Krebs cycle metabolites indicates overall Krebs cycle activity resulting from D_7_-Glc and ^13^C_5_,^15^N_1_-Gln oxidation, respectively.

**Figure 2:**
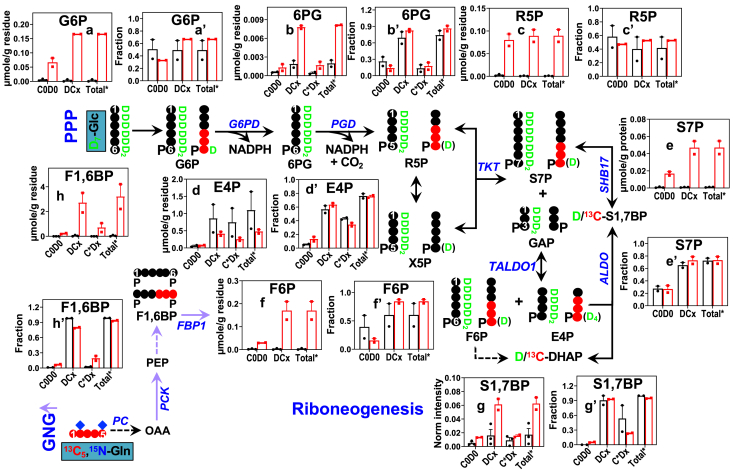
**CA PD-OTC of CZ019 activates the pentose phosphate pathway, gluconeogenesis, and riboneogenesis.** The same polar extracts in Figure 1 were analyzed for the D, ^13^C, and ^15^N labeling patterns of metabolites in the PPP, GNG, and riboneogenesis pathways to reconstruct reprogramming of CA *versus* NC PD-OTC. Labeled species are as described in Figure 1. See Table S3 for statistics. 6PG, 6-phosphogluconate; ALDO, aldolase; DHAP, dihydroxyacetone-3-phosphate; E4P-erythrose-4-phosphate; F1,6BP, fructose-1,6-bisphosphate; F6P, fructose-6-phosphate; FBP1, fructose bisphosphatase 1; G6P, glucose-6-phosphate; G6PD, glucose-6-phosphate dehydrogenase; PC, pyruvate carboxylase; PCK, phosphoenol pyruvate carboxykinase; PGD, phosphogluconate dehydrogenase; R5P, ribose-5-phosphate; SHB17, sedoheptulose-bisphosphatase; S7P, sedoheptulose-7-phosphate; S1,7BP, sedoheptulose-1,7-bisphosphate; TALDO1, transaldolase 1; TKT, transketolase; UGP2, UDP-Glucose pyrophosphorylase 2.

**Figure 3:**
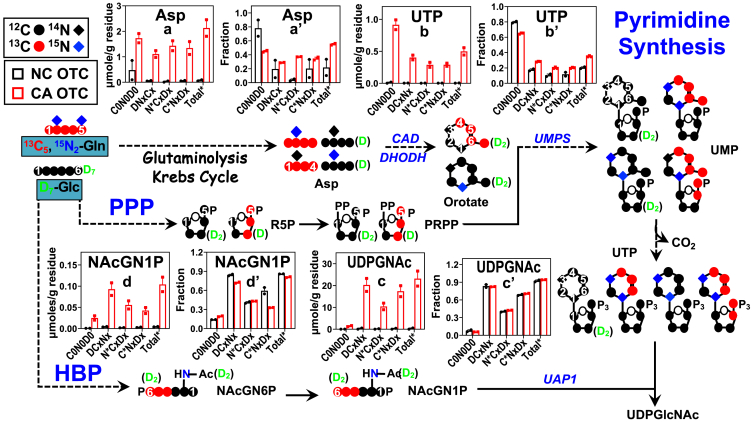
**CA PD-OTC activates synthesis of pyrimidine/sugar nucleotides.** The same polar extracts in Figure 1 were analyzed for the D, ^13^C, and ^15^N labeling patterns of metabolites in the the metabolic pathway of pyrimidine and sugar nucleotides to reconstruct reprogramming of CA *versus* NC OTC. Labeled species and all other abbreviations are as described in Figure 1. See Table S3 for statistics. DHODH, dihydroorotate dehydrogenase; HPB: hexosamine biosynthesis pathway; PRPP, phosphoribosyl pyrophosphate; NAcGN1/6P, N-acetylglucosamine-1/6-phosphate; UAP1, UDP-N-acetylglucosamine pyrophosphorylase 1; UDPGNAc, UDP-N-acetylglucosamine, CAD, carbamoyl-phosphate synthetase 2, Aspartate transcarbamylase, and dihydroorotase; UMPS, Uridine monophosphate synthetase.

Relative to NC OTC, the CA OTC of CZ019 exhibited enhanced glutaminolysis, which fueled increased GSH biosynthesis as evidenced by the increase in the level and fractional enrichment of ^13^C,^15^N-bearing Glu (a-a’), ^13^C_5_-αKG (b-b’), and ^13^C,^15^N-bearing GSH (c-c’) (Fig. 1*B*). The Krebs cycle was also activated in CA *versus* NC OTC, which was indicated by enhanced conversion of labeled αKG (Total∗; b-b’) to labeled succinate, fumarate, malate, and citrate (Total∗; d-g and d’-g’). However, the fractional enrichment of labeled succinate (Total∗; d’) and fumarate (e’) was lower than that of labeled malate (f’) and citrate (g’) in CA OTC. These data suggest a reversal of the latter half of the Krebs cycle reactions from citrate to malate, which raised the question as to the source of labeled citrate. We found elevated level and fractional enrichment of ^13^C_5_-citrate and ^13^C_3_-malate in CA *versus* NC OTC, which points to enhanced RC as the source of labeled citrate. The two labeled species could only be produced *via* the react+ion sequence of RC-ATP citrate lyase (ACLY)-malate dehydrogenase (MDH)1 when the canonical Krebs cycle activity was disrupted at SDH (*cf.* Fig. 1*B* scheme). In addition, we saw increased buildup and fraction of ^13^C_2_- (C2Dx)/^13^C_3_-bearing (C3Dx) citrate in CA *versus* NC OTC, which suggests enhanced activity of MDH1-ME1-ME2/PC and/or malate/Asp shuttle (mitochondrial malate import and Asp export, not shown)-ME2/PC. Moreover, the elevated level and fractional enrichment of ^13^C_1_-bearing (C1Dx) malate agree with enhanced ME2 activity *via* the generation of ^13^C_1_-pyruvate in the reverse direction. The changed trend of D-bearing (DCx, DCxNx) Krebs cycle metabolites in CA *versus* NC OTC was akin to that of their ^13^C-bearing counterparts (C∗Dx, C∗NxDx). This further supports a broken Krebs cycle at succinate in CA OTC, leading to RC and enhanced production of ME/PC reaction products.

A similar reprogramming of glutaminolysis, GSH synthesis, and the Krebs cycle was evident in the CA OTC of CZ020, CZ021 (Fig. S2), and CZ016 (Fig. S3) but not in the CA OTC of CZ017 (Fig. S4) and CZ022 (Fig. S5). It should be noted that CZ017 had a different tumor subtype (ER^low+^, PR^-^, and HER2^-^) from the other 5 patients (*cf.* Fig. S1*H*) and had undergone neoadjuvant therapy prior to surgery, while CZ022’s tumor had a significant fraction of fatty tissues. These data suggest that isocitrate dehydrogenase 1/2 and ME1/2 inhibitors might be effective in some but not all BC.

### Variable activation of PPP and GNG in the CA OTC of BC patients

Other than glycolysis, glucose is a major precursor for the PPP, which converts glucose to ribose-5-phosphate (R5P) *via* the oxidative branch (PPP_ox_) and interconverts R5P, sedoheptulose-7-phosphate, glyceraldehyde-3-phosphate, erythrose-4-phosphate (E4P), and fructose-6-phosphate *via* the nonoxidative branch (PPP_non-ox_) (Fig. 2 scheme). A noncanonical PPP pathway (RNG) in yeast (31) can also generate R5P from dihydroxyacetone-3-phosphate (glycolytic and/or gluconeogenic product) and E4P *via* aldolase (ALDO) and transketolase activity. This pathway bypasses NADPH production in case of high demand for ribose during ribosome biogenesis (31). Gln is not a usual precursor for these pathways unless GNG is active.

We saw enhanced conversion of D_7_-glucose to 6-phosphogluconate (b-b’), a key D bearing (DCx) PPP_ox_ metabolite and R5P (c-c’) in CA *versus* NC OTC of CZ019 (Fig. 2), which indicates increased activity of PPP_ox_ and production of NADPH in CA OTC. CA OTC also showed activated PPP_non-ox_, as evidenced by elevated level and comparable fractional enrichment of D-bearing sedoheptulose-7-phosphate (e-e’) and fructose-6-phosphate (f-f’). However, neither the level nor fractional enrichment (d-d’) of D-bearing E4P was elevated in CA *versus* NC OTC, which suggests the influence of additional pathway(s). These data, together with the production of D-seduheptulose-1,7-bisphosphate (S1,7BP; g-g’) points to active RNG, which to the best of our knowledge is a novel finding in mammalian systems. The higher fractional enrichment of D-bearing S1,7BP than that of its one precursor D-bearing E4P is presumably attributed to the expectedly high enrichment of the other precursor D-bearing dihydroxyacetone-3-phosphate produced from glycolysis. We also found significant levels of ^13^C-bearing (C∗Dx) 6-phosphogluconate, fructose-1,6-bisphosphate (F1,6BP), and S1,7BP in CA *versus* NC OTC, which can only be produced from ^13^C_5_,^15^N_2_-Gln *via* GNG. Activated GNG and RNG in the CA OTC of CZ019 is evident by enhanced buildup and higher or comparable enrichment of their key products, ^13^C bearing F1,6BP (h-h’) and D bearing S1,7BP (Fig. 2*G*-g’), respectively in CA *versus* NC OTC. Activation of PPP and RNG was also evident in the CA *versus* NC OTC of CZ016, 20 to 22 (Figs. S3, S5, and S6). However, we did not see evidence for the activation of PPP/GNG/RNG in the CA OTC of CZ017 (Fig. S4). These data suggest that both PPP and RNG blockers would be required to inhibit R5P production needed for nucleotide synthesis in BC.

### Activation of pyrimidine, purine, and sugar nucleotide turnover in the CA OTC of BC patients

Both glucose and Gln can be major fuel sources for the synthesis of pyrimidine nucleotides *via* the production of the Krebs cycle metabolite Asp and the PPP metabolite R5P/phosphoribosyl pyrophosphate (PRPP) (15), as shown in the scheme of Figure 3. The pyrimidine nucleotide product UTP in turn serves as the precursor to the synthesis of UDP-N-acetylglucosamine (UDPGNAc), a key metabolite for supporting N- and O-linked glycosylation of proteins, which play important roles in cell-cell recognition, growth, differentiation, programmed cell death, and cancer development including metabolic reprogramming (32, 33, 34).

In both CA and NC OTC of CZ019, we detected D, ^15^N, and ^13^C labeling of UTP (b-b’) and UDPGNAc (c-c’) as well as their synthetic intermediates Asp (a-a’) and N-acetylglucosamine-1-phosphate (NAcGN1P) (d-d’) (Fig. 3), which confirms *de novo* synthesis of UTP and UDPGNAc from both glucose and Gln, as expected. CA OTC showed enhanced labeling of UTP compared to NC OTC, which suggests activated synthesis. Increased labeling of NAcGN1P (d-d’) and UDPGNAc (c-c’) was also evident, which points to the activation of the hexosamine biosynthesis pathway. We further saw enhanced buildup of unlabeled NAcGN1P with no significant change of the fractional enrichment, which indicates enhanced synthesis of unlabeled NAcGN1P in CA *versus* NC OTC. The unlabeled precursors could have originated from the degradation of pre-existing glycogen and/or unlabeled glucogenic amino acids. The latter is consistent with enhanced GNG (*cf.* increased ^13^C labeling of F1,6BP in Fig. 2) from unlabeled source(s) in CA *versus* NC OTC of CZ019. A similar activation of hexosamine biosynthesis pathway and UTP/UDPGNAc synthesis from glucose and Gln was evident for the CA *versus* NC OTC of CZ020 and 021, as inferred from similar change trend in the labeling patterns of UTP, NAcGN1P, and UDPGNAc (Fig. S7, *A*b–d and *B*b’, c’, d') in CA *versus* NC OTC. Moreover, CA OTC of CZ016 and 022 showed none or relatively less enhanced D, ^13^C, or ^15^N labeling of UTP and UDPGNAc, when compared with the NC counterparts (Figs. S3 and S5). However, D, ^15^N, and ^13^C labeling of NAcG1P was enhanced in the CA *versus* NC OTC of CZ022 (Fig. S5) but attenuated in CA *versus* NC OTC of CZ016 (Fig. S3). Thus, CA OTC of different patients bearing the same BC subtypes (CZ016, CZ019–22) (*cf.* Fig. S1*H*) share common but also distinct reprogramming of pyrimidine nucleotide and UDPGNAc metabolism.

**Figure 4:**
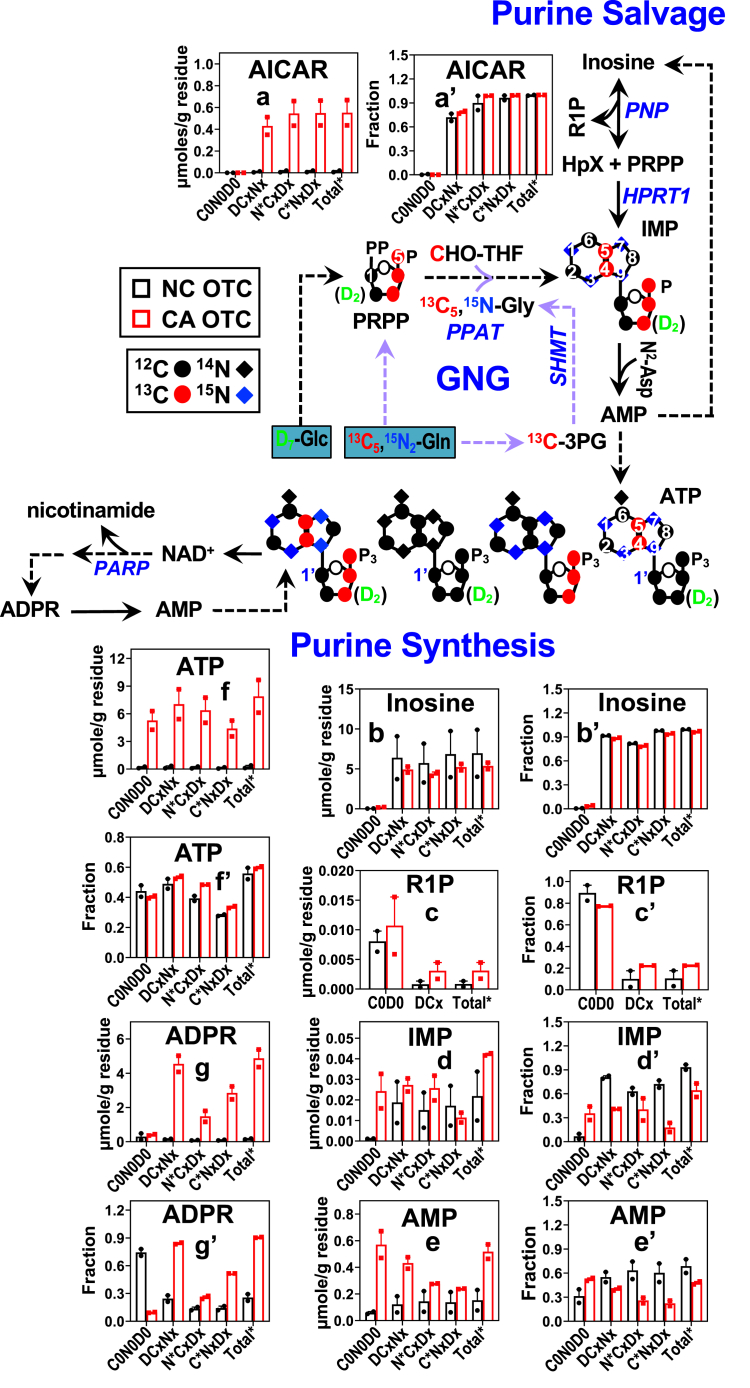
**CA PD-OTC activates *de novo*/salvage synthesis of purine nucleotides and ADP-ribosylation.** The same polar extracts in Figure 1 were analyzed for the D, ^13^C, and ^15^N labeling patterns of metabolites in the metabolic pathways of purine nucleotides to reconstruct reprogramming of CA *versus* NC OTC. Labeled species and all other abbreviations are as described in Figure 1. See Table S3 for statistics. 3PG: 3-phosphoglycerate; CHO-THF: 10-Formyltetrahydrofolate; AICAR: 5-aminoimidazole-4-carboxamide ribonucleotide; R1P: ribose-1-phosphate; HpX: hypoxanthine; Nic: nicotinamide; SHMT: serine hydroxymethyltransferase; PPAT: phosphoribosyl pyrophosphate amidotransferase; PNP: purine nucleoside phosphorylase; HPRT1: hypoxanthine phosphoribosyltransferase 1; NAMPT: nicotinamide phosphoribosyltransferase; PARP: poly(ADP-ribose) polymerase.

For the synthesis of purine nucleotides, both *de novo* and salvage pathways can be involved (Fig. 4). Glycine and PRPP are precursors to the purine ring and ribose subunits, respectively, which are readily produced from glucose *via* glycolysis, the serine-glycine-one carbon pathway, and PPP, but not from Gln unless GNG is active. Gln is the source of purine ring nitrogen either directly from the amido group or indirectly from the amino group. The *de novo* synthesis pathway generates the intermediate 5-aminoimidazole-4-carboxamide ribonucleotide (AICAR) while the salvage pathway produces inosine (degradation product of AMP) and ribose-1-phosphate. The two pathways converge at inosine monophosphate (IMP), which is the substrate for both ATP and GTP production. ATP in turn generates NAD^+^, which is the substrate for ADP ribosylation (*e.g.* poly(ADP-ribosyl)ation or PARylation) of proteins. This PTM regulates a number of biological processes including genomic stability, inflammation, energy metabolism, apoptosis, and signal transduction, all of which are important to cancer development (35). Turnover of PARylated proteins generates ADP-ribose (ADPR), which can be hydrolyzed to produce AMP to support salvage synthesis of ATP (36). Thus, AMP and IMP production is modulated by a complex interplay of the *de novo* and salvage purine synthesis pathways and protein PARylation/turnover pathways.

For all OTC of the six patients, we observed both *de novo* and salvage synthesis of purine nucleotides from D_7_-glucose and ^13^C_5_,^15^N_2_-Gln. This is evidenced by D, ^15^N, and ^13^C labeling of the intermediates such as AICAR, IMP, inosine, and ribose-1-phosphate, as well as the products ATP and ADPR (Figs. 4, S3–S5, and S8). Except for CZ017, CA OTC exhibited a higher level with comparable fractional enrichment of labeled AICAR relative to NC OTC (Figs. 4*A*, a’, S3, S5, *A*v and *B*v’, and S8, *A*a, h and *B*a’, h’). In addition, all CA OTC exhibited enhanced labeling of ATP (Figs. 4*F*, f’, S3–S5, *A*aa, and *B*aa’, and S8, *A*m and *B*m’), which points to activated *de novo* synthesis of purine nucleotides. We also saw largely a higher fractional enrichment of D- (DCxNx), ^15^N- (N∗CxDx), and ^13^C-bearing (C∗NxDx) inosine (Figs. 4*B*’, S3–S5*B*w’, and S8*B*c’, j’) than that of the IMP (Figs. 4*D*’, S3–S5*B*y’, and S8b’, i’) and AMP (Figs. 4*E*’, S3–S5*B*z’, and S8*B*e’, l’) counterparts in all OTC. This indicates that inosine was not only a product of IMP/AMP catabolism but also a salvage precursor *via* the action of purine nucleoside phosphorylase (PNP)-HPRT (Fig. 4 scheme). CA OTC showed largely depletion of D-, ^15^N-, and ^13^C-bearing inosine with comparable or reduced fractional enrichment relative to NC OTC, which suggests increased flow from labeled inosine to IMP over label replenishment *via* AMP degradation, that is, more activated salvage synthesis of ATP than its replenishment from AMP degradation. Further noted for most patients was the enhanced buildup of unlabeled (C0N0D0) IMP/AMP but not inosine with increased or comparable fractional enrichment in CA *versus* NC OTC (Figs. 4, S3–S5, and S8), which points to additional input into the IMP/AMP but not the inosine pool from unlabeled sources(s) such as preexisting glycogen and/or glucogenic essential amino acids. Suffice to say, both glucose and Gln-fueled synthesis of ATP was greatly enhanced in CA *versus* NC OTC of all six patients. This could support enhanced production of ADPR (*i.e.* buildup of Total∗) *via* NAD^+^-fueled ADP ribosylation and turnover. Together, these data suggest that blocking the synthesis of purine nucleotides could be an effective strategy for BC therapy.

### mSIRM coupled with protein profiling by reverse phase protein arrayRPPA informs translational dysregulation in the CA OTC of BC patients

The mSIRM approach in essence provided simultaneous *in situ* assays for numerous metabolic pathways in BC patient tissues. The reprogrammed pathways discerned could be caused by the altered expression of key metabolic proteins in CA patient tissues. We thus utilized reverse phase protein array (RPPA) to quantify the expression of many of these protein candidates deduced from the mSIRM analysis. RPPA is a protein profiling method that requires only low nL of protein lysates with high quantitative precision, sensitivity, and sample throughput (37, 38). These merits make RPPA much better suited for our needs than standard protein analysis methods such as Western blotting and ELISA. We also quantified the PTM version of selected protein targets.

**Figure 5:**
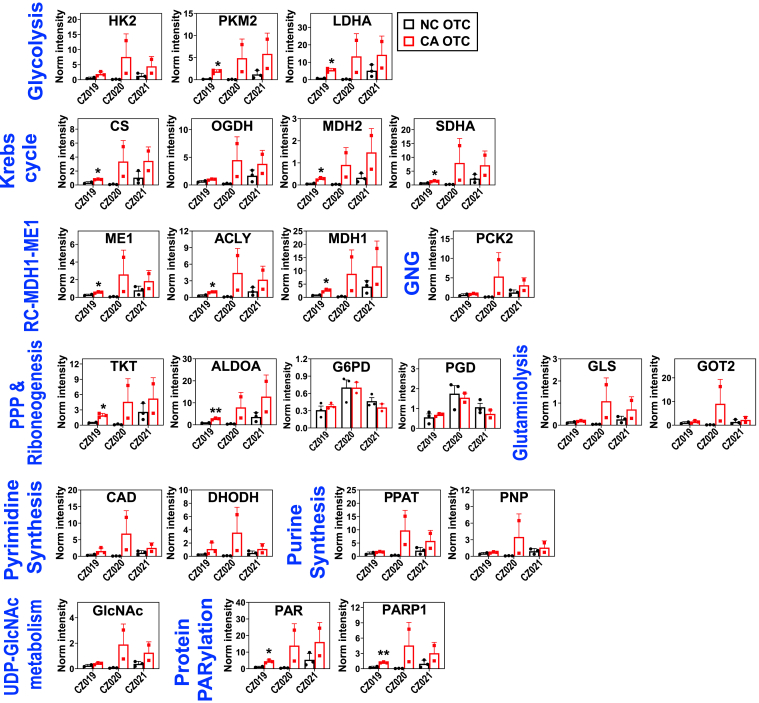
**Expression changes in key metabolic proteins in part account for reprogrammed metabolic network in CA PD-OTC of CZ019-021.** Relevant metabolic proteins in activated metabolic networks in Figure 1, Figure 2, Figure 3, Figure 4 were profiled in the corresponding protein extracts using reverse phase protein array (RPPA) as described in Experimental procedures. All other abbreviations are as described in Figure 1, Figure 2, Figure 3, Figure 4. See Table S6 for statistics. PKM2: pyruvate kinase M2; PAR: PARylated proteins; GlcNAc: O-linked glycosylated proteins.

In general, replicate CA OTC showed more variable expression of central metabolic proteins than replicate NC OTC (Fig. 5), which is presumably due to greater cellular heterogeneity in the CA tissue. This variability often compromised the statistical significance of the expression differences even between paired CA and NC OTC. Although statistically insignificant in many cases, we could relate differential expression of key metabolic proteins to reprogrammed metabolism in CA OTC. For example, we saw overexpression of glycolytic enzyme hexokinase 2, pyruvate kinase M1/2, and lactate dehydrogenase A in CZ019-021’s CA OTC (Fig. 5), which is consistent with activated glycolysis. Overexpression of citrate synthase, MDH2, oxoglutarate dehydrogenase, and succinate dehydrogenase (SDHA) in these CA OTC may contribute to the enhanced conversion of citrate to succinate *via* the canonical Krebs cycle and to malate *via* the RC–ACLY–ME1 pathway (*cf.* Fig. 1*B*). The latter is also consistent with their overexpression of ME1, ACLY, and MDH1 (Fig. 5). We saw overexpression of transketolase and ALDOA in the CA OTC of 019-021 (Fig. 5), which can lead to enhanced R5P synthesis *via* PPP_non-ox_ and RNG (*cf.* Fig. 2 scheme). Overexpression of glutaminase and glutamate oxaloacetate transaminase 2 (GOT2) in the CA OTC of CZ020 (Fig. 5) could account for the highly activated metabolism from Gln to αKG (Fig. S2). Likewise, overexpression of PCK2 in this OTC (Fig. 5) can be linked to activated GNG (Fig. S6).

Activated R5P synthesis coupled with overexpression of CAD (carbamoyl-phosphate synthetase 2, aspartate transcarbamylase, and dihydroorotase) and dihydroorotate dehydrogenase in the CA OTC of CZ019-021 can drive enhanced synthesis of pyrimidine nucleotides in these tissues. We also saw enhanced expression of O-GlcNAcylated proteins (GlcNAc) in these OTC, which is presumably driven by activated UDP-GlcNAc synthesis (Figs. 3 and S7). Enhanced O-GlcNAcylation of key oncoproteins has been shown to promote BC growth and resistance to anti-estrogen therapy (39). Likewise, increased synthesis of purine nucleotides in the CA OTC of CZ020 can be driven by overexpression of phosphoribosyl pyrophosphate amidotransferase and PNP (Fig. 5), which are respectively key enzymes in the *de novo* and salvage synthesis pathways of purine nucleotides ((40); Fig. 4 scheme). Moreover, we noted the overexpression of PARylated proteins (PAR) and poly(ADP-ribose) polymerase 1 in the CA OTC of CZ019-021 (Fig. 5), which points to enhanced PARylation as deduced from enhanced labeling of ADPR (*e.g.* Fig. 4). Poly(ADP-ribose) polymerase 1 is a major enzyme that plays a crucial role in DNA damage repair *via* PARylation of target proteins and has been shown to mediate antiestrogen resistance in human BC cells (41).

It should be noted that protein expression data did not always directly relate to the reprogrammed metabolic activity ascertained from mSIRM analysis. For example, none of the CA OTC showed enhanced expression of glucose-6-phosphate dehydrogenase and 6-phosphogluconate dehydrogenase (Fig. 5), suggesting that PPP_ox_ activation is not regulated at the protein level. It could be mediated *via* substrate, NADPH/NADP^+^ ratio, and/or allosteric regulation of these enzymes, which has been reported (42, 43). Similarly, the expression of CAD and dihydroorotate dehydrogenase did not differ between CA and NC OTC of CZ019/021 (Fig. 5), which suggests that activated UTP synthesis in CA OTC (Figs. 3, S7) is not driven by the expression of these two enzymes, but some other factors such as phosphorylation or substrate level control (44). Thus, CA OTC of different BC patients shared common reprogramming of many pathways, but the underlying mechanism for metabolic dysregulation can differ among them. Such knowledge is crucial to developing effective metabolic target(s) for BC therapy.

### Inhibition of PCK blocks purine nucleotide synthesis while eliciting tissue damage in CA OTC

Since SIRM tracing suggested preferential diversion of Gln-derived GNG products to fuel the synthesis of purine nucleotides, we asked whether blocking GNG interfere with the latter activity, thus compromising BC growth. 3-MPA is known to inhibit PCK activity (9), which initiates GNG. We recovered CA and matched NC breast OTC of CZ017 from cryopreservation and treated them with 1 mM 3-MPA for 20 days with the addition of D_7_-Glc and ^13^C_5_,^15^N_2_-Gln tracers for the last 46 h. As for OTC of freshly resected tissues, both CA and NC OTC exhibited tissue outgrowth after a total of 36 days of culturing with the last 20 days under 3-MPA or no treatment (Fig. S9*A*). NC OTC appeared to show more significant outgrowth in response to 3-MPA while CA OTC did not in two out of three replicates. Based on H&E-stained images of the main tissues, 3-MPA treatment led to reduced cellularity for CA OTC but not for NC OTC (Fig. S9*B*). This was accompanied by increased levels of total (CASP3) and cleaved caspase 3 in two out of three 3-MPA–treated *versus* control main tissues (M) of CA OTC but not in their outgrowth (OG) or NC counterparts. Again, tissue heterogeneities among replicates were evident, which presumably contributed to the variable responses to 3-MPA.

**Figure 6:**
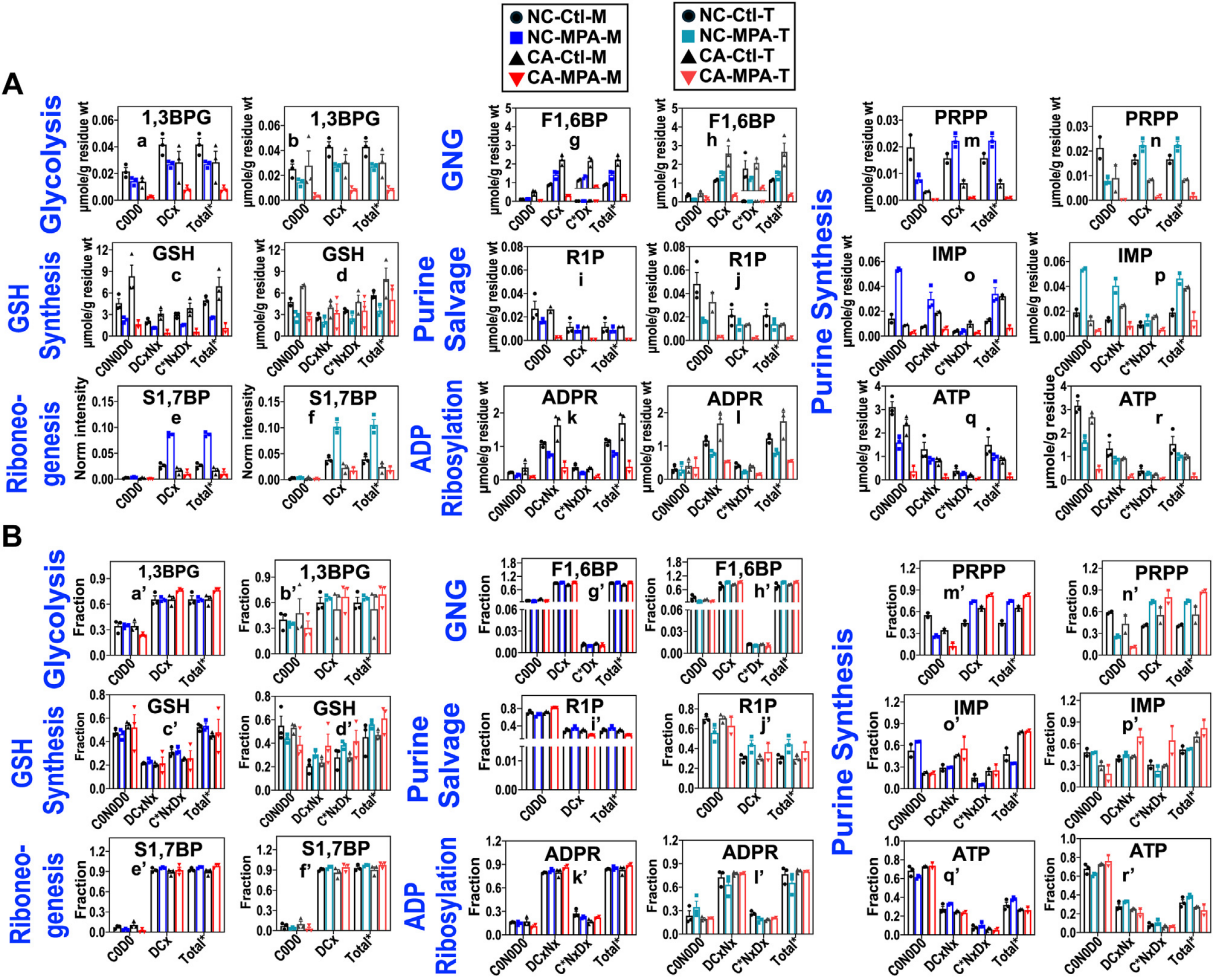
**PCK inhibition blocks glycolysis, synthesis of purine nucleotides, and ADP-ribosylation in CA PD-OTC of CZ017.** The tracer experiment and PCKi 3-MPA (1 mM) treatment of cryorevived CA and NC PD-OTC for CZ017 patient-bearing ER^low^, PR^-^, and HER2^-^ IDC was performed as described in Experimental procedures (n = 2–3 biological replicates). The SIRM data were presented as μmole/g residue (*A*) and fraction (*B*) for the main tissue (MPA-M) and sum of main tissue + outgrowth (MPA-T). Metabolites shown are key indicators of central catabolic and anabolic pathways altered by 3-MPA. Labeled species and all other abbreviations are as described in Figure 1, Figure 2, Figure 3, Figure 4. See Table S7 for statistics. – : Ctl; : 3-MPA-treated main tissue of NC OTC; : 3-MPA-treated main tissue of CA OTC; : 3-MPA-treated main tissue + outgrowth of NC OTC; : 3-MPA-treated main tissue + outgrowth of CA OTC; 1,3BPG: 1,3-bisphosphoglycerate.

We also saw differential effects of 3-MPA on Glc and Gln-fueled metabolic networks, as illustrated in Figure 6, where the labeled level (A) and fractional enrichment (B) of key markers of reprogrammed metabolic pathways were shown for the main tissue (M; Ctl , ; 3-MPA ,) *versus* main tissue plus outgrowth combined or total (T; Ctl , ; 3-MPA ,). As expected, PCK inhibition by 3-MPA reduced ^13^C incorporation into GNG products F1,6BP in CA main tissues or main + outgrowth tissues, as evidenced by depletion of its ^13^C isotopologs (C∗Dx, g-h) (A) without changes in fractional enrichment (referred as reduced ^13^C labeling thereafter) (g’-h’, B). This was accompanied by reduced ^13^C labeling of IMP, ATP, and ADPR (Ao-r, k-l; Bo’- r’, k’-l’), which indicates reduced incorporation of Gln carbon into purine nucleotides *via de novo* and/or salvage synthesis pathways. 3-MPA treatment did not elicit these changes or to a lesser extent in the NC counterparts.

Likewise, PCK inhibition reduced D (DCx) incorporation into glycolytic intermediates 1,3-BPG (Aa-b) and F1,6BP (Ag-h) (Fig. 6), which suggest attenuated glycolysis by 3-MPA in CA main tissues and main + outgrowth tissues. Again, this did not occur in the NC counterparts. 3-MPA also blocked GSH synthesis in CA main tissues but not in main + outgrowth tissues, as evidenced by the changes of their D- and ^13^C-labeling patterns (Ac-d; Bc’-d’). Moreover, we saw no difference in the D- and ^13^C-labeling patterns of S1,7BP in 3-MPA treated *versus* control CA OTC (Ae-f; Be’-f’), which suggested little impact of 3-MPA treatment on RNG in CA OTC. However, a markedly enhanced D incorporation into S1,7BP was induced by 3-MPA in the NC counterparts, which points to increased capacity for RNG. Finally, 3-MPA attenuated D labeling of metabolites PRPP, IMP, and ATP for CA OTC but not for NC OTC (Fig. 6 Am-r; Bm’-r’). Together, these data suggest that 3-MPA reduced the flow of both glucose and gluconeogenic Gln carbon into purine nucleotides in CA but not in NC OTC. Such differential effects of 3-MPA on RNG and synthesis of purine nucleotides could account for more outgrowth and better maintenance of tissue cellularity of NC OTC than CA OTC (Fig. S9). Thus, PCK1/2 could be a promising target for BC therapy.

We then asked if 3-MPA–induced metabolic reprogramming was mediated by altered expression of relevant metabolic proteins. Profiling of key metabolic proteins by RPPA in Fig. S9*C* revealed suppression of lactate dehydrogenase A by 3-MPA in both CA and NC OTC (c-d), which is consistent with decreased lactic fermentation observed by SIRM analysis. We also saw overexpression of phosphofructokinase platelet in CA OTC and its suppression in NC OTC (a-b), which is inconsistent with attenuated glycolysis in the former. However, phosphofructokinase platelet suppression has been shown to support BC cell survival and epithelial mesenchymal transition *via* shunting glucose-6-phosphate into the PPP (45), which is consistent with the maintenance of cellularity and more significant tissue outgrowth seen in NC OTC (Fig. S9, *A* and *B*). 3-MPA also elicited PCK2 buildup in NC OTC but either not or less significantly in CA OTC (Fig. S9*C*e, f). This could reflect a feedback induction of PCK2 expression to overcome its inhibition by 3-MPA, leading to insignificant block of GNG-fueled purine nucleotide synthesis and ADP ribosylation in NC OTC (Fig. 6). In contrast, 3-MPA reduced the FBP1 level in the main tissues (, ) of NC OTC while enhancing it in the CA counterparts (Fig. S9*C*g). This could counteract the effect of PCK2 overexpression in NC OTC but could also lead to inhibition of glycolysis and epithelial mesenchymal transition in CA OTC (13). Furthermore, 3-MPA differentially (mainly oppositely) altered protein expression in other metabolic pathways in CA *versus* NC OTC (Fig. S9*C*). Statistically significant overexpression in CA or suppression in NC main tissues included proteins in Gln metabolism (i, k, o), PPP (q), and purine synthesis (u) (Fig. S9*C*). Overexpression of these proteins in CA main tissues points to enhanced Gln uptake/metabolism and synthesis of purine nucleotides, which is contrary to the SIRM data (Fig. 6). Also noted was the lack of overexpression in ALDOA (Fig. S9*C*s–t) in NC OTC, which is inconsistent with the enhanced incorporation of D labels into S1,7BP (Fig. 6*A*e and f). These inconsistencies raise the likelihood of PTM and/or altered allosteric regulation of these proteins by 3-MPA. They could also be partly offset by GOT2 suppression (although nonstatistically significant) in 3-MPA–treated *versus* control CA OTC (Fig. S9*C*o and p), which would restrict Gln entry into the Krebs cycle and subsequent metabolism into purine nucleotides. However, the above protein changes (except for GOT2 overexpression) are consistent with reduced Gln metabolism and purine synthesis in NC OTC (Fig. 6). Also consistent with the SIRM data of IMP (Fig. 6, o and p) and regulation of its synthesis *via* the salvage pathway is 3-MPA–induced suppression of PNP in the CA OTC and its overexpression in the NC OTC ( *versus* , w;  *versus* , x) (Fig. S9*C*). The complex dysregulation of these metabolic proteins by 3-MPA informs new strategies for BC patient therapy including patients (*e.g.* CZ017) who fail the neoadjuvant therapy. For example, combining glutaminase or phosphogluconate dehydrogenase with PCK inhibition could synergize anticancer action.

## Discussion

Our novel long-term culturing method coupled with mSIRM approach employed in this study provides unequivocal evidence for the reprogramming of extensive metabolic networks in human BC tissues, which cannot be obtained without tracers or even with single tracer-based metabolomics approaches. Nor could the knowledge be unambiguously gained from transcriptomic or proteomic analysis. These included enhanced noncanonical Krebs cycle (notably Gln-driven reductive carboxylation/ME and PC-mediated anaplerosis), preferential diversion of GNG products to purine/pyrimidine synthesis, and NAD^+^-mediated PARylation/turnover of PARylated proteins. It also enabled a direct comparison of metabolic utilization of glucose and Gln in pathways critically important to cancer cell survival and development, without suffering from sample batch artifacts. Sample batch variations can be significant in patients’ tissues due to cellular heterogeneities and variable TME interactions (*e.g.* Fig. S9*B*; lung cancer OTC (46)). The matched CA *versus* NC design allows individual patient tumor’s metabolic reprogramming and response to drugs to be interrogated. Furthermore, an important advantage of the mSIRM approach is the efficient use of very limited patient specimens, whether they be resected tissues or patient-derived organoids. The novel reprogrammed events learned from mSIRM analysis of BC patient cancer tissues not only advance our understanding of BC functions but can also provide effective target(s) for therapy.

It is now commonly recognized that activated glycolysis and anaplerosis of the Krebs cycle enhance growth of cancer cells (18, 47, 48). The occurrence of RC is also known to support cancer cell growth by sustaining the production of anabolic Krebs cycle metabolites in cancer cells under hypoxia (49) or in cells with a defective Krebs cycle such as FH-mutated renal carcinoma cells (50). Activated RC in the CA OTC of all six BC patients could be a result of disrupted Krebs cycle at the SDH site (*cf.* Fig. 1*B*). However, SDHA protein expression was not attenuated in CA *versus* NC OTC (Fig. 5). It is possible that SDHA is inactivated allosterically and/or *via* PTM, which awaits further investigation.

To support excess growth, CA tissues will need to produce NADPH and R5P to fuel lipid and nucleotide synthesis, respectively. NADPH is also required to regenerate GSH for defense against oxidative stress. The D labeling patterns of the PPP metabolites are consistent with increased production of NADPH and *de novo* synthesis of R5P *via* PPP_ox_ and PPP_non-ox_ in CA *versus* NC OTC, except for CZ017’s OTC (Figs. 2 and S3–S6). Whether the differential behavior of CZ017’s OTC is related to its distinct tumor subtype, more advanced stage, and/or the influence of neoadjuvant therapy (Fig. S1*H*) requires further studies.

Regardless of the tumor genotype or stage, Gln-fueled GNG was activated in BC OTC to drive nucleotide synthesis. However, we found GNG to be absent in the 2D culture of MCF-7 cells but active in that of ZR-75-1 cells, as evidenced by ^13^C_5_-Gln–fueled production of ^13^C-glycogen, ^13^C-Gly, and ^13^C-1′-ribose of adenine nucleotides (AXP) in ZR-75-1 cells (Fig. S10 and unpublished results). This is in contrast to the GNG activity reported for MCF-7 cells in a recent study, which was inferred from the scrambled labeling patterns of ^13^C_6_-glucose–derived pyruvate, Ser, and Gly (11). Such an inference is ambiguous as other metabolic activity including PPP can also lead to scrambling of the ^13^C labels in these metabolites. MCF-7 and ZR-75-1 are respectively ER^+^/PR^+^/HER2^-^ and ER^+^/PR^+^/HER2^+^ ductal carcinoma of luminal A subtype (51, 52). Based on our previous findings, the two cell lines also had differential metabolic network activity as *in vitro* 2D cell cultures *versus in vivo* tumor xenograft (14). These differences could reflect distinct tumor genotype(s) such as the *HER2* expression status and/or the influence of TME, which is absent in 2D cell cultures. The influence of TME on tumor metabolic reprogramming could also be inferred from activated GNG-fueled synthesis of purine nucleotides in all ER^+^/PR^+^/HER2^-^ CA OTC, which was absent from 2D MCF-7 cells with the same genotype. Whether this influence is due to reprogrammed metabolism of cancer cells and/or that of other cell types in the TME such as cancer-associated fibroblasts await further studies.

Consistent with preferential flow of glucogenic Gln carbon into purine nucleotides, PCK inhibitor 3-MPA blocked this process by attenuating GNG in CZ017’s CA OTC but not in NC OTC (Fig. 6). Presumably, NC OTC is more resistant to 3-MPA in GNG inhibition and such differential effects of 3-MPA contribute to better outgrowth and maintenance of tissue cellularity in NC *versus* CA OTC (Fig. S9). Likewise, attenuated effect of 3-MPA on glycolysis and GSH synthesis as well as its stimulating effect on RNG (Fig. 6) can also contribute to the insensitivity of NC OTC to 3-MPA treatment. Better maintenance of GSH synthesis could also contribute to the resistance of CA OTC’s outgrowth to 3-MPA as GSH synthesis was much less attenuated in the main + outgrowth than the main tissues (Fig. 6).

**Figure 7:**
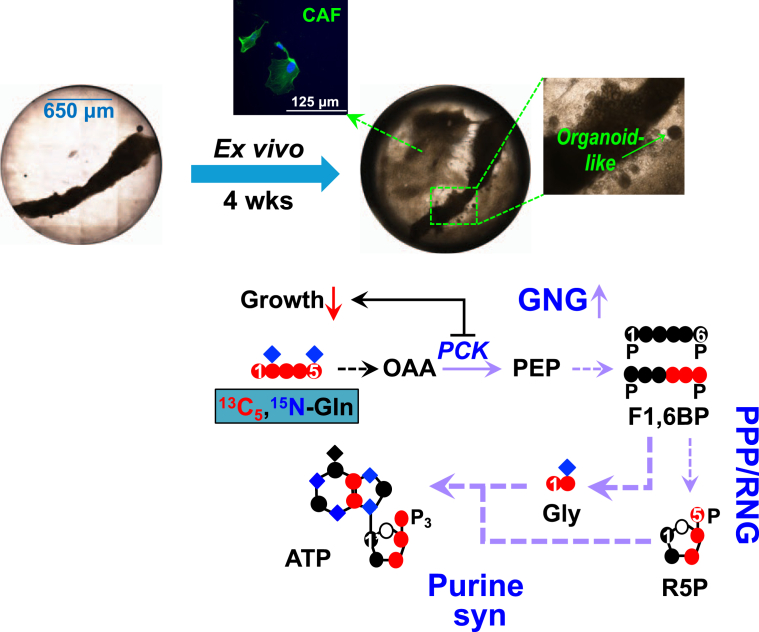
***Ex vivo* culturing coupled with SIRM studies uncovers preferential diversion of Gln-fueled GNG products for purine synthesis as distinct metabolic features common to all breast cancer PD-OTC.** Our long-term *ex vivo* culturing method maintained cell and metabolic viability of CA OTC derived from breast cancer patients for at least 4 weeks. It enabled isolation of cancer-associated fibroblasts (CAF) and organoids. ^13^C_5_,^15^N_2_-Gln-based SIRM study of cultured PD-OTC revealed Gln-fueled GNG plus riboneogenesis (RNG) and preferential diversion of the products to support adenine nucleotide (AXP) synthesis as a distinct metabolic feature common to all six CA-OTC. Blocking GNG by PCK inhibition led to reduced tumor tissue growth.

In conclusion, our culturing method enabled maintenance and growth of both fresh and cryopreserved patient-derived breast tissues *ex vivo* for over a month. These PD-OTC displayed extensive metabolic activity and outgrowth with CA OTC harboring isolatable organoids and fibroblasts. Interrogation of reprogrammed metabolism in CA *versus* NC OTC with dual ^2^H_7_-glucose and ^13^C_5_,^15^N_2_-Gln tracers coupled with SIRM analysis revealed novel reprogrammed events such as RNG and preferential diversion of GNG products to fuel the synthesis of purine nucleotides. In particular, the latter event is common to all BC tissues (Fig. 7). When blocking this process with PCK inhibitor 3-MPA, metastatic, ER^low^/PR^-^/HER2^-^ CA OTC displayed compromised cellularity, reduced outgrowth, and growth/survival-supporting metabolism but matched NC OTC did not. Thus, PD-OTC with native patient tissue architectures and microenvironment represent unique models for elucidating reprogramming of individual BC patient’s tumor metabolism to uncover viable metabolic targets such as PCK while enabling target testing for evaluating therapeutic efficacy and resistance mechanism.

## Experimental procedures

### Patient accrual and tissue processing

BC patients (CZ016-017, 019-022) were consented under the approved protocol of the University of Kentucky Institutional Review Board (IRB#44224, Total Cancer Care) for their tissue collection prior to surgery (see Fig. 1*G* for patient attributes). Tissue processing was as described previously (28). Briefly, resected CA and matched NC tissues were embedded in 3% low-melting agarose before cutting into 750 μm slices using a Krumdieck tissue slicer (Alabama Research & Development). For CZ016-017 and 020-022, tissue slices were cultured fresh for metabolic studies while the rest were cryopreserved by placing in STEM-CELLBANKER (#11924, Amsbio) before freezing at −80 °C and storing in liquid N_2_ freezer. For CZ019, tissue slices were revived from cryopreservation by quick warming at 37 °C before culturing.

### *Ex vivo* organotypic tissue culturing

Each tissue slice (fresh or cryopreserved, 15–40 mg wet weight) was embedded in Matrigel (as Matrigel:BC organoid medium or BCOM at 1:1 ratio; see Table S1 for composition (53)) on a 0.4 μm Biopore membrane insert (#PICM0RG50, MilliporeSigma) and placed in a well of a 6-well plate containing 2 ml of BCOM (*cf.* Fig. S1*A*). The slice cultures (OTC) were incubated at 37 °C/5% CO_2_ with gentle rocking for 1 to 1.5 months with periodic medium change and microscopic examination. Medium samples were collected for ^1^H NMR analysis for glucose/Gln uptake and lactate release.

### Dual stable isotope tracer treatment

Each patient’s OTC was replaced with a stable isotope tracer medium 2 to 3 weeks after culturing in BCOM. The tracer medium was composed of 0.68% (w/v) D_7_ (^2^H_7_)-glucose + 7.44 mM ^13^C_5_,^15^N_2_-Gln in BCOM and the treatment lasted for 2 (CZ016-017) or 3 days (CZ019-022). At tissue harvest, two small pieces of CZ016-017 OTC were cut for histology and live fluorescence spectroscopy while the rest were metabolically quenched in liquid N_2_ before further processing for SIRM analysis. The CZ019-022 OTC were processed similarly for histology and SIRM analysis.

### Fluorescence spectroscopy of live OTC

Live CZ016 and CZ017 breast OTC were treated with a glucose analog 2-NBDG or TMRE (an indicator of mitochondrial membrane potential) before fluorescence was acquired as described in Yan *et al.*, 2023 (28). Briefly, a small piece was cut from each OTC at the end of SIRM experiment, washed with PBS, and incubated at 37 °C, 5% CO_2_ for 60 min in 200 μmol/L 2-NBDG or 50 nmol/L TMRE dissolved in glucose-free BCOM. The tissues were then rinsed with PBS before fluorescence spectroscopic assay to report glucose uptake and mitochondrial metabolism.

### Tissue and medium extraction

Frozen OTC were pulverized into 10 μm particles in liquid N_2_ with a SPEX 6775 Freezer/Mill (SPEX SamplePrep) to maximize metabolite extraction. Polar/nonpolar metabolites and proteins were extracted simultaneously using the acetonitrile:H_2_O:chloroform (2:1.5:1, v/v) solvent partitioning method as described previously (16). Polar metabolite extracts were split and lyophilized for NMR and IC-UHRFTMS analysis described below. For medium extraction, a 50 μL aliquot was mixed with 200 μL cold acetone (−20 °C) (80% acetone final) and kept at −80 °C for 20 to 30 min before centrifugation at 21,100*g* for 20 min at 4 °C to remove protein precipitates. The extracts were lyophilized before NMR analysis.

### SIRM analysis

Lyophilized polar extracts were dissolved in 35 L D_2_O containing 17.5 nmol DSS for 1D ^1^H and ^1^H{^13^C} heteronuclear single quantum coherence (HSQC) NMR measurement or in H_2_O for anionic ion chromatography coupled with ultra high-resolution Fourier transform mass spectrometric (IC-UHRFTMS) analysis (see below). The D-, ^13^C-, and/or ^15^N-labeling patterns (total abundance in μmole/g residue and fractional enrichment) of relevant metabolites were used to reconstruct various catabolic and anabolic pathways (46).

### NMR spectroscopy

As described previously (26), ^1^H NMR spectra were recorded at 15 °C on either an Agilent DD2 14.1 T spectrometer equipped with a 3 mm HCN triple resonance cold probe or a Bruker Avance III 16.45 spectrometer equipped with a 1.7 mm HCN triple resonance cryoprobe using an acquisition time of 2 s and a relaxation delay of 4 s with weak presaturation on the residual HOD peak. 1D ^1^H{^13^C}-HSQC spectra were recorded at 15 °C with an acquisition time of 0.2 s and a relaxation delay of 1.8 s with adiabatic ^13^C decoupling. Free induction decays were transformed using Mnova (Mestrelab Research), with zerofilled to 128 k complex points and a 0.5 Hz line-broadening exponential (^1^H) or zerofilled to 16 k complex points and a 4 Hz line broadening exponential (HSQC). After Fourier transformation, phasing, baseline correction, and referencing to internal DSS, resonances were assigned using our in-house database (54) and integrated using the Mnova line fitting as a mixed Lorentz-Gaussian line shape. Peak integral for each spectrum were corrected for the number of protons, calibrated against DSS to determine the absolute amount, and then normalized to tissue residue weight to obtain metabolite content as μmol/g residue. The fractional enrichment of site-specific ^13^C, F was calculated from the ^1^H spectra as:

F = A(^13^C)/[A(^13^C)+A(^12^C)]

where A(^13^C) and A(^12^C) are the peak areas of the proton directly attached to ^13^C and ^12^C, respectively.

### IC-FTMS

IC-UHR-FTMS was performed as described previously (55). Briefly, metabolites were separated on a Thermo Scientific Dionex IonPac AG11-HC analytical column paired with a Dionex IonPac AS11-HC guard column in a Dionex ICS-5000^+^ DP ion chromatography system. Eluted metabolites were detected by a Thermo Fisher Orbitrap Fusion Tribrid mass spectrometer run in both negative MS^1^ and MS/MS (data-independent analysis) modes. The MS^1^ scan was acquired with a resolving power of 500,000 at *m/z* 200 and an *m/z* range of 80 to 800, while the data-independent analysis scan was obtained with a resolution of 60,000 and a precursor *m/z* range of 280 to 440 with a wide isolation window of 200 m/z. TraceFinder software and an in-house database were used for data analysis. MS^1^ peak areas of assigned metabolites and isotopologs were corrected for natural abundance and quantified against a calibration standard mixture. Metabolite amounts thus obtained were normalized by dry residue weight.

### Isotopologue nomenclature

As three stable isotopes were used, the number of transformed isotopologs was too large and unnecessary to display individually. For example, Asp with four ^13^C, three ^15^N, and one D (C_4_N_3_D) may have up to 5∗2∗4 = 40 isotopologs. We thus summed up relevant isotopologs for each metabolite for meaningful interpretation. For example, C3Dx is the sum of isotopologs having three ^13^C atoms and any number of D, whereas C∗Dx is the sum of isotopologs with ≥ one ^13^C and any number of D. Total∗ is the sum of all labeled isotopologs observed. The unlabeled isotopolog is C0N0D0, that is, no ^13^C, ^15^N, or D. Further details are given in the figure legends.

### RPPA analysis

RPPA analysis was performed as described previously (19). Briefly, protein extracts (at ≤ 0.8 mg/ml) from the tracer experiments were printed as spots onto a slide coated with nitrocellulose membrane pads (Oncyte SuperNOVA 16 NC pads, Grace Bio-Labs) using a microarray printer (ArrayJet, Ltd). Printed slides were first stained for total protein using an Azure RPPA staining kit (#AC2233, VWR Scientific) and scanned at 700 nm emission wavelength using an InnoScan 710 AL Microarray Scanner (Innopsys, Inc). Slides were then immunoblotted in ELISA grade primary antibodies at suitable dilutions (see Table S2) overnight at 4 °C, followed by incubation in host-matched fluorescent secondary antibodies (AzureSpectra 800 Conjugates) at 1:8000 dilution and room temperature for 1 h and scanned at 800 nm emission wavelength with InnoScan 710 AL. All antibodies used were validated by vendors *via* negative response to protein knockdown/knockout, induction in response to stress (anti-PAR antibody), and/or single band in Western blot. Protein fluorescence and immunofluorescence for the same protein spot were done using the Innopsys Mapix software. Both fluorescence intensities were corrected for background before normalizing the immunofluorescence intensity with the protein fluorescence intensity.

### Immunofluorescence staining and microscopy

Cancer-associated fibroblast-like cells (400 cells/well) from Fig. S1*G* were seeded in a μClear flat-bottom black 384-well plate (#781091, Greiner Bio-One) and cultured in Dulbecco’s modified Eagle’s medium supplemented with 10% fetal bovine serum and 1X penicillin-streptomycin antibiotics for 6 days. Cells were then fixed in 4% paraformaldehyde in PBS for 10 min, washed three times with PBS, permeabilized in PBS containing 0.1% Triton X-100 for 10 min, washed three times with PBS, incubated overnight at 4 °C in primary antibodies against ACTA2 (αSMA), VIM, FAP, or COL1A1 (see Table S2), washed three times with PBS, incubated in anti-rabbit Alexa594 (#A11012) or anti-mouse Alexa647 secondary antibody (#A21235) (Invitrogen) at 1:2000 dilution for 1 h, then in PBS + 0.5 μg/mL DAPI, and washed three times in PBS before imaging using an EVOS M7000 fluorescence microscope (Thermo Fisher Scientific). After four weeks of culturing, CZ019’s OTC were subsampled, fixed in 10% formalin, embedded in paraffin wax, and sectioned into 4 μm slices. The formalin-fixed paraffin-embedded sections were deparaffinated and subjected to antigen retrieval before immunofluorescence staining for Ki67 (MKI67) and ER (*cf.* Table S2) using vendor’s protocol for FlexAble Antibody Labeling Kits (Proteintech Group), that is, rabbit IgG-based FlexAble Coralite 555 and 647 protocol for Ki67 and ER, respectively. Fluorescent images were obtained using a VS120 slide scanner (Olympus).

## Ethics approval and consent to participate

All patients were consented prior to surgery for their tissue collection under the approved protocol of the University of Kentucky Institutional Review Board (IRB#44224, Total Cancer Care). The studies in this work abide by the Declaration of Helsinki principles.

## Data availability

Except for patient-derived specimens and demographic data, all materials and data are available upon reasonable request. Summary data are provided in the Figures and supporting information. Statistical analyses are provided in supplementary data files. Patient-derived materials will require materials transfer agreement. Patient demographic/pathologic data are protected by IRB protocol #44224. Relevant data for this manuscript have been deposited at Dryad, https://doi.org/10.5061/dryad.4qrfj6qnm.

## Supporting information

This article contains supporting information.

## Conflict of interest

The authors declare that they have no conflicts of interests with the contents of this article.
