# Supplementary material for: Patient-derived organotypic tissue cultures as a platform to evaluate metabolic reprogramming in breast cancer patients
Source: J Biol Chem. 2025 Apr 8;301(5):108495. doi: 10.1016/j.jbc.2025.108495 (PMC12137166; doi:10.1016/j.jbc.2025.108495)
Supplement: Supporting materials [file mmc1.pdf]

Supporting Information

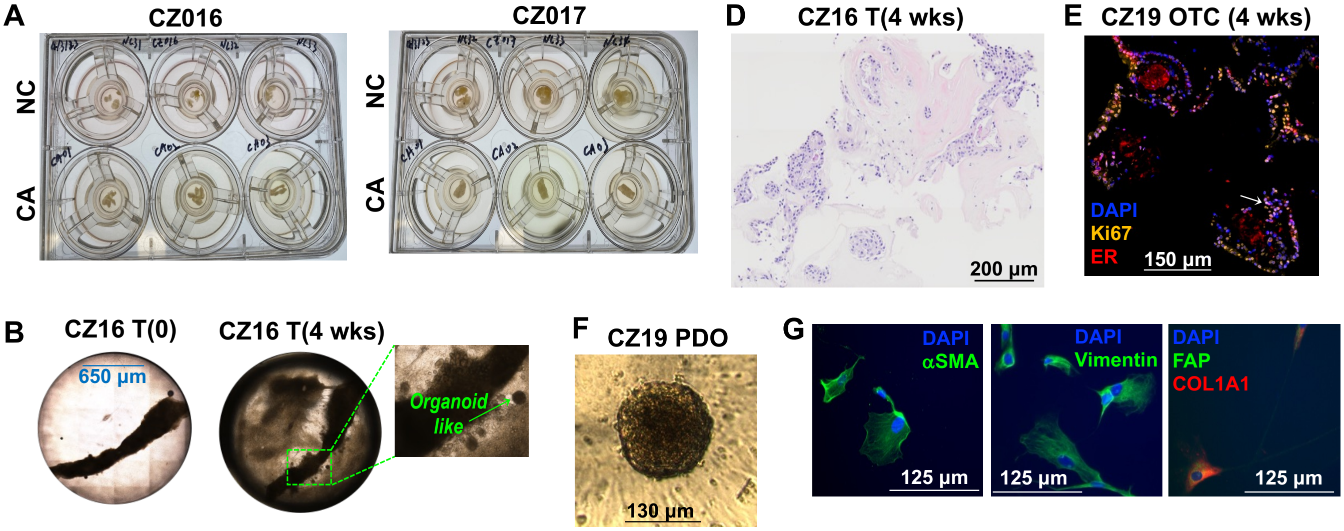

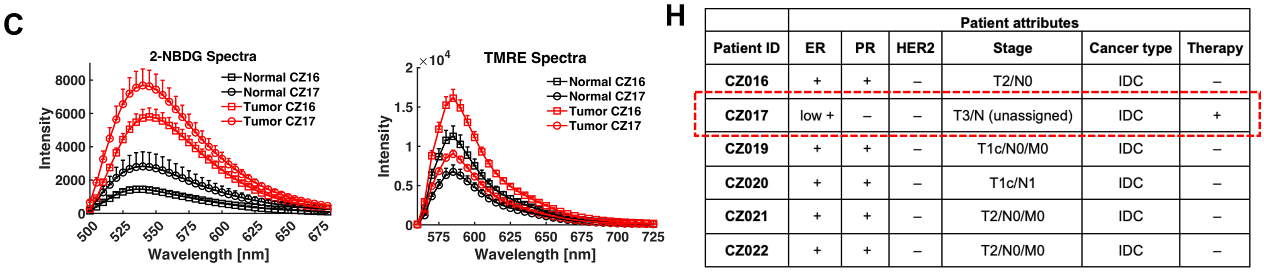

H

	Patient attributes					
Patient ID	ER	PR	HER2	Stage	Cancer type	Therapy
CZ016	+	+	–	T2/N0	IDC	–
CZ017	low +	–	–	T3/N (unassigned)	IDC	+
CZ019	+	+	–	T1c/N0/M0	IDC	–
CZ020	+	+	–	T1c/N1	IDC	–
CZ021	+	+	–	T2/N0/M0	IDC	–
CZ022	+	+	–	T2/N0/M0	IDC	–

Figure S1. CA PD-OTC show increased glucose uptake/mitochondrial membrane polarization & outgrowth with isolatable organoids and fibroblasts.
Fresh breast tissues of CZ016/017 and cryopreserved breast tissues of CZ019 were cultured as described in Methods and Materials. CA OTC of CZ016 and 017 showed enhanced acidification compared with NC OTC after 4 wks. of culturing (**A**). At harvest, extensive tissue outgrowth with organoids and fibroblast-like structures was evident as illustrated in **B** for CZ16 CA OTC. Live fluorescence spectroscopic analysis showed increased uptake of glucose (2-NBDG as marker) and mitochondrial membrane potential (TMRE as marker) in CA versus NC OTC of CZ016 and 017 (**C**, [Reprinted/Adapted] with permission from [ref 28] © Optical Society of America). H&E stain displayed structural integrity of cultured CZ016's CA OTC (**D**) and immunofluorescent stain showed the abundance of nuclear ER^{+}/$Ki67^{+}$ cells (e.g. at arrow) in cultured CZ019's CA OTC (**E**). Patient-derived organoid (PDO) was isolated from CZ019's CA OTC and established in culture (**F**). Cancer-associate fibroblast isolated from CZ017 OTC stained positive for fibroblast markers α-smooth muscle actin (αSMA or ACTA2), vimentin (VIM), fibroblast activation protein alpha (FAP), and Collagen Type I Alpha 1 Chain (COL1A1) (**G**). Patient and tumor attributes for the six BC-PD-OTC studied were listed in **H**.

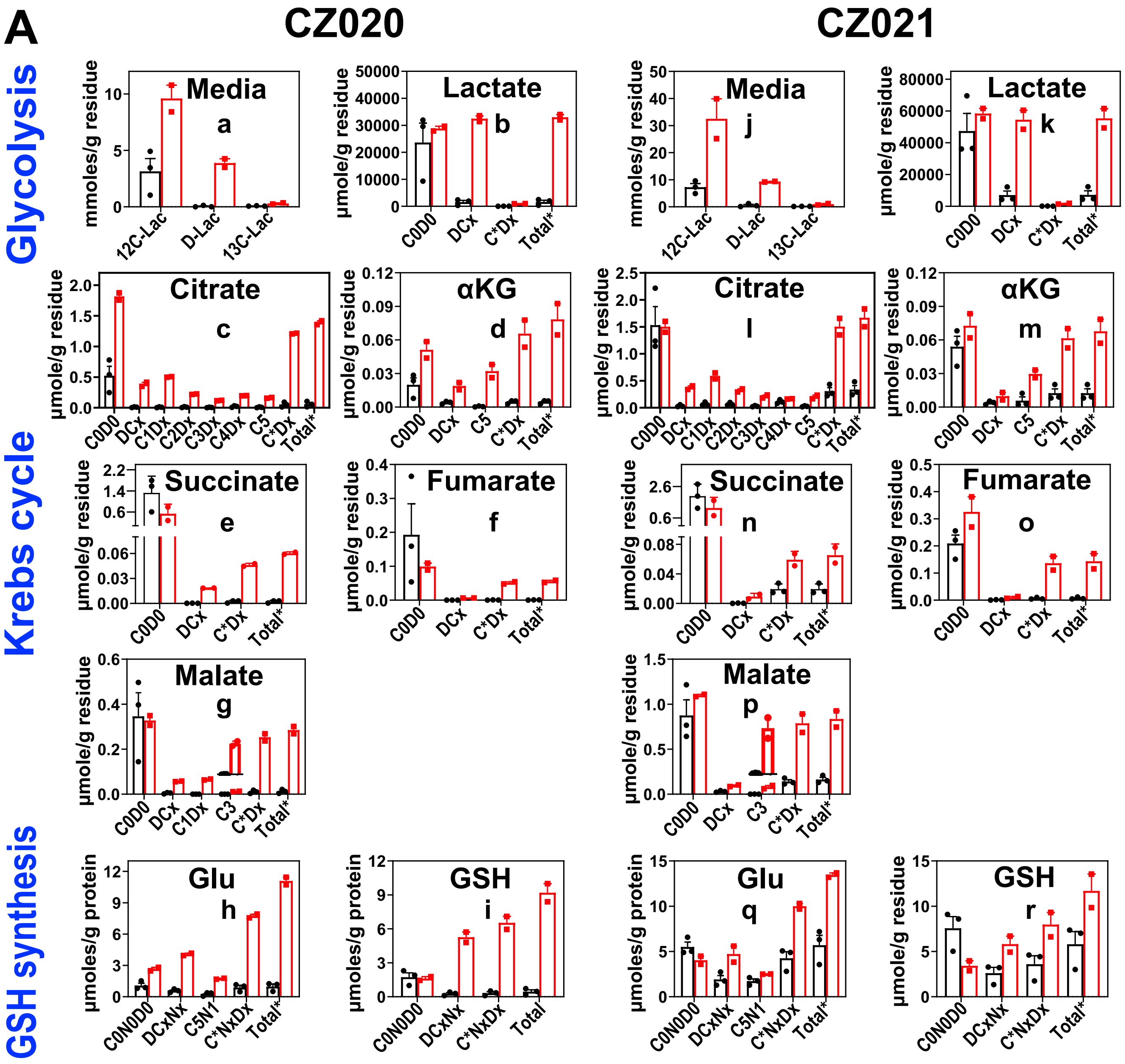

A
CZ020
CZ021
Glycolysis
Krebs cycle
GSH synthesis
Media
a
Lactate
b
Media
j
Lactate
k
Citrate
c
αKG
d
Citrate
l
αKG
m
Succinate
e
Fumarate
f
Succinate
n
Fumarate
o
Malate
g
Malate
p
Glu
h
GSH
i
Glu
q
GSH
r
mmoles/g residue
µmole/g residue
µmoles/g protein
µmole/g residue
12C-Lac
D-Lac
13C-Lac
C0D0
DCx
C*Dx
Total*
C1Dx
C2Dx
C3Dx
C4Dx
C5
C3
C0N0D0
DCxNx
C5N1
C*NxDx
Total

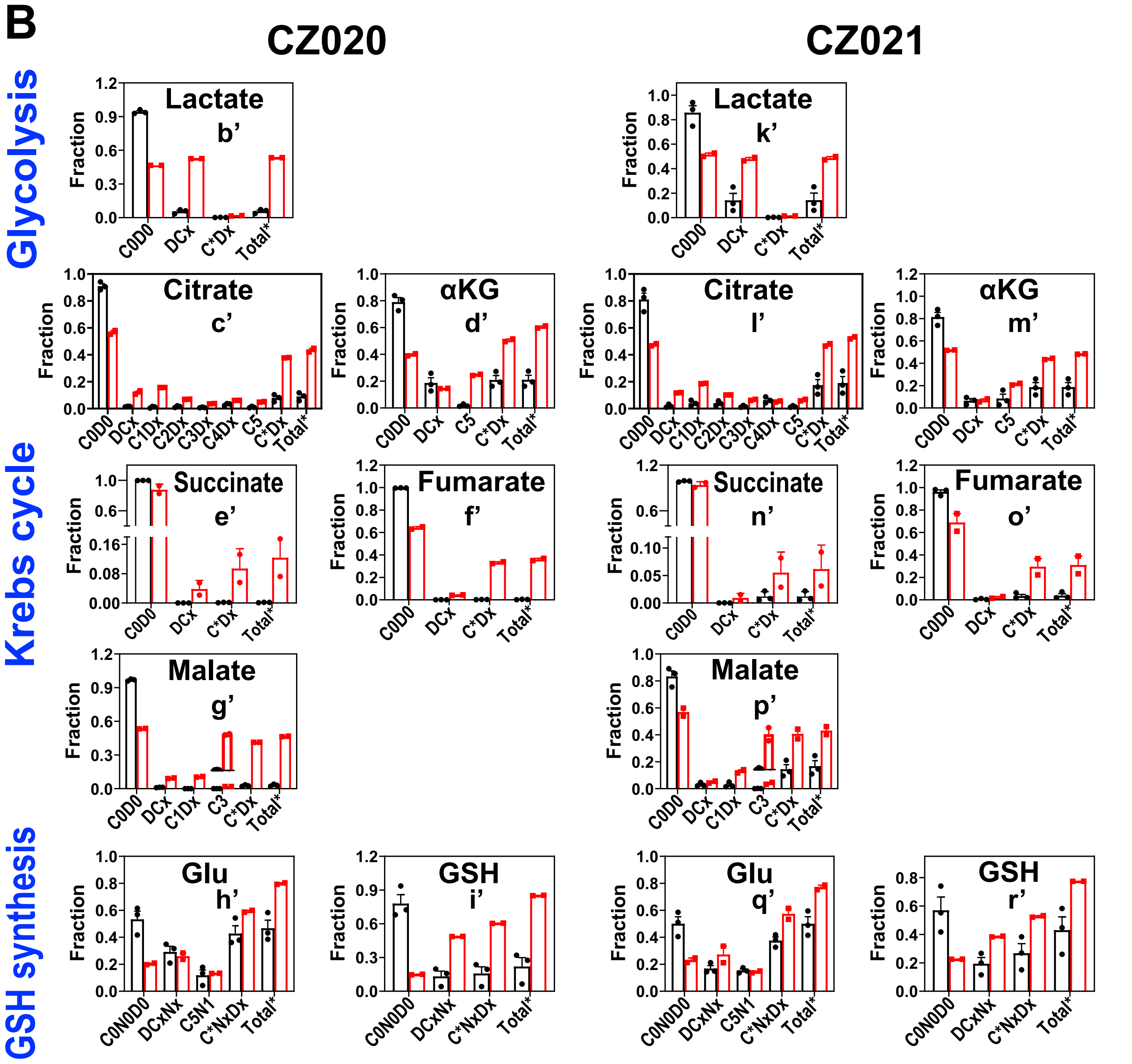

Figure S2. CA PD-OTC of CZ020-021 displays enhanced glycolysis and non-canonical Krebs cycle metabolism.

Freshly prepared CA (■) and NC (●) OTC of CZ020-021 were cultured and subjected to tracer treatment as in **Fig. 1** (n = 2-3 biological replicates). The SIRM data were presented as μmole/g residue (**A**) and fraction (**B**). All symbols and abbreviations are as in **Fig. 1**. See **Table S4** for statistics.

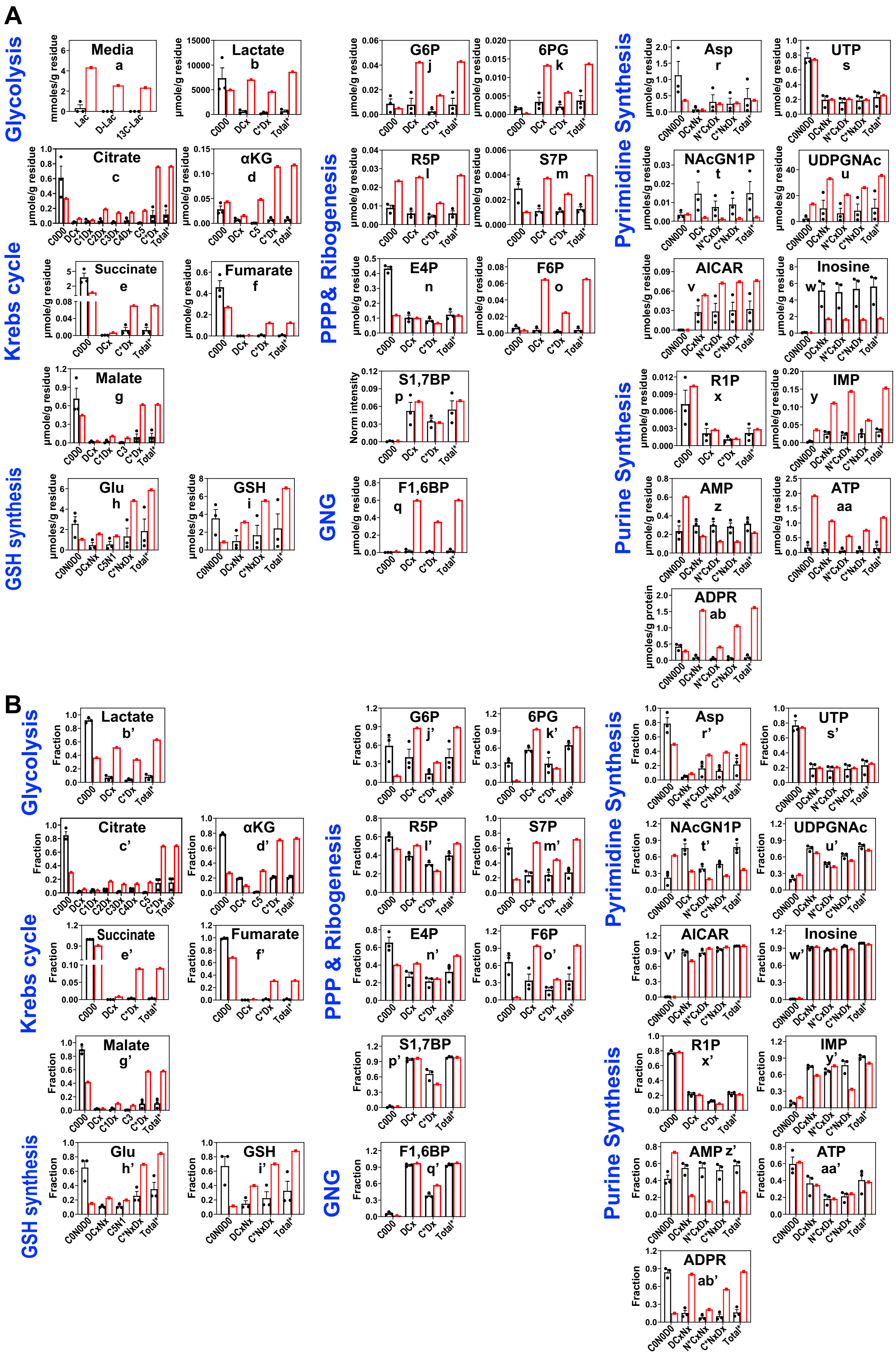

Figure S3. CA PD-OTC of CZ016 displays enhanced glycolysis, non-canonical Krebs cycle metabolism, PPP, GNG, purine nucleotide synthesis, and ADP ribosylation.

Freshly prepared CA (■) and NC (●) OTC of CZ016 were cultured and subjected to tracer treatment as in **Fig. 1** (n = 1 or 3 biological replicates). The SIRM data were presented as µmole/g residue (**A**) and fraction (**B**). All symbols and abbreviations are as in **Figs. 1-4**. No statistical analysis was done as n = 1 for CA OTC.

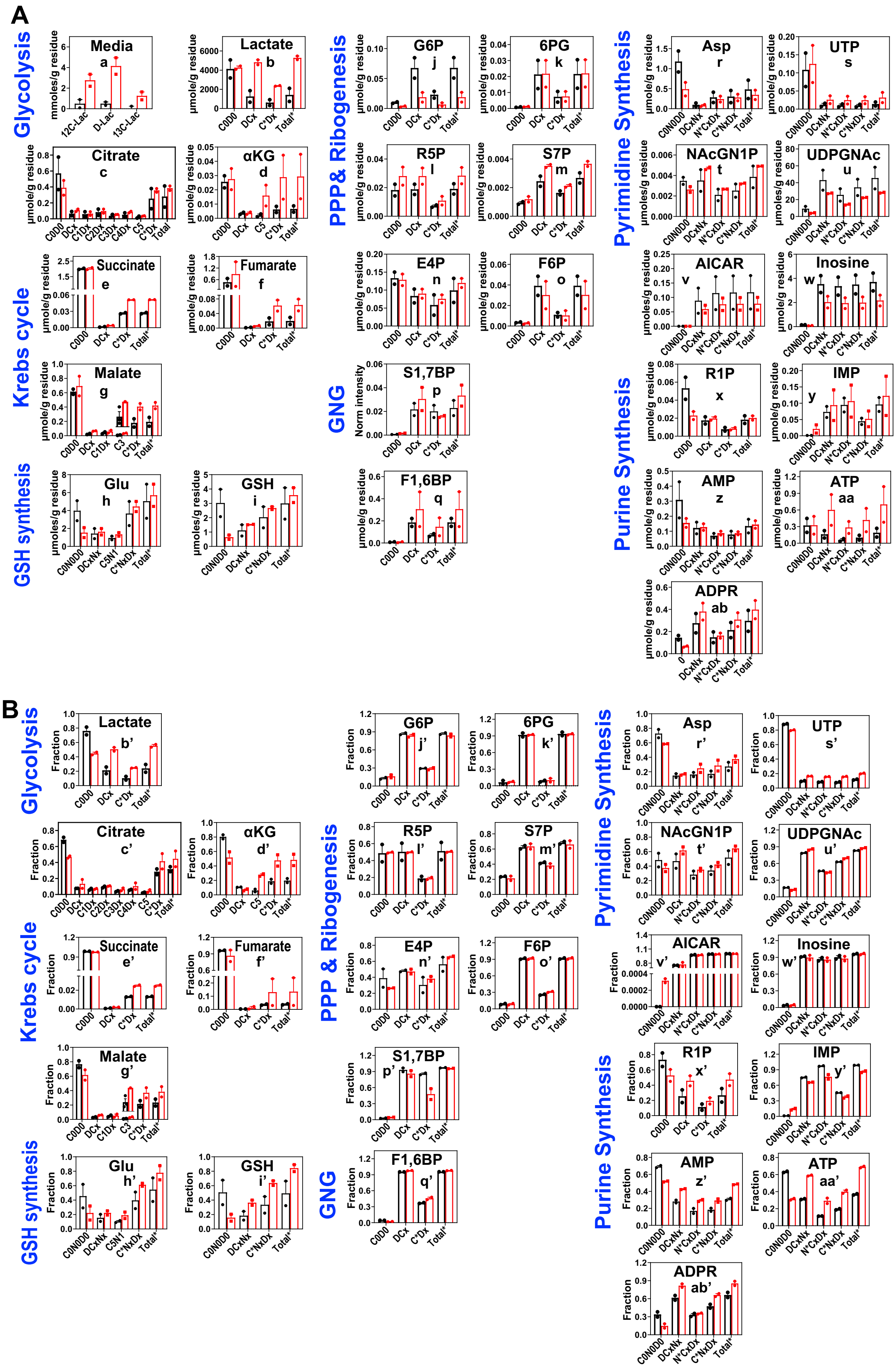
A
Glycolysis
Media a
mmoles/g residue
12C-Lac
D-Lac
13C-Lac
Lactate b
µmole/g residue
C0D0
DCx
C*Dx
Total*
PPP& Ribogenesis
G6P j
6PG k
Pyrimidine Synthesis
Asp r
UTP s
C0N0D0
DCxNx
N*CxDx
C*NxDx
Citrate c
C1Dx
C2Dx
C3Dx
C4Dx
C5
Total
αKG d
R5P l
S7P m
NAcGN1P t
UDPGNAc u
Krebs cycle
Succinate e
Fumarate f
E4P n
F6P o
AICAR v
Inosine w
Malate g
C3
GNG
S1,7BP p
Norm intensity
Purine Synthesis
R1P x
IMP y
GSH synthesis
Glu h
C5N1
C*NxDx
GSH i
F1,6BP q
AMP z
ATP aa
ADPR ab
B
Glycolysis
Lactate b'
Fraction
G6P j'
6PG k'
Pyrimidine Synthesis
Asp r'
UTP s'
Citrate c'
αKG d'
PPP & Ribogenesis
R5P l'
S7P m'
NAcGN1P t'
UDPGNAc u'
Krebs cycle
Succinate e'
Fumarate f'
E4P n'
F6P o'
AICAR v'
Inosine w'
Malate g'
S1,7BP p'
Purine Synthesis
R1P x'
IMP y'
GSH synthesis
Glu h'
GSH i'
GNG
F1,6BP q'
AMP z'
ATP aa'
ADPR ab'

Figure S4. CA PD-OTC of CZ017 displays enhanced glycolysis, Krebs cycle metabolism, and turnover of purine nucleotides/ADP ribosylated proteins.

Freshly prepared CA (■) and NC (●) OTC of CZ017 were cultured and subjected to tracer treatment as in **Fig. 1** (n = 2 biological replicates). The SIRM data were presented as μmole/g residue (**A**) and fraction (**B**). All symbols and abbreviations are as in **Figs. 1-4**. See **Table S5** for statistics.

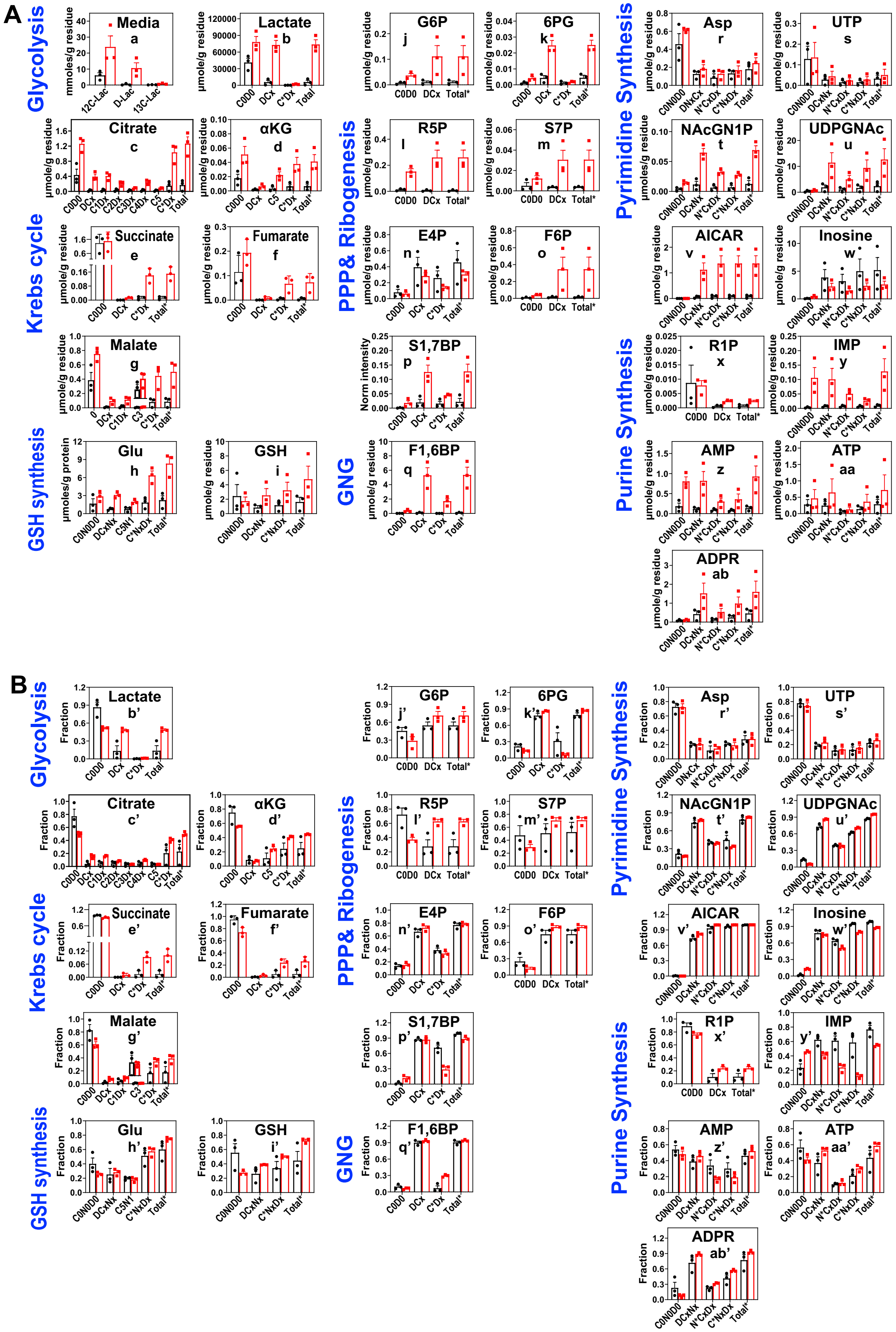

A
Glycolysis
Krebs cycle
GSH synthesis
PPP& Ribogenesis
GNG
Pyrimidine Synthesis
Purine Synthesis
Media a
Lactate b
Citrate c
αKG d
Succinate e
Fumarate f
Malate g
Glu h
GSH i
G6P j
6PG k
R5P l
S7P m
E4P n
F6P o
S1,7BP p
F1,6BP q
Asp r
UTP s
NAcGN1P t
UDPGNAc u
AICAR v
Inosine w
R1P x
IMP y
AMP z
ATP aa
ADPR ab
mmoles/g residue
µmole/g residue
µmoles/g residue
µmoles/g protein
Norm intensity
B
Lactate b'
Citrate c'
αKG d'
Succinate e'
Fumarate f'
Malate g'
Glu h'
GSH i'
G6P j'
6PG k'
R5P l'
S7P m'
E4P n'
F6P o'
S1,7BP p'
F1,6BP q'
Asp r'
UTP s'
NAcGN1P t'
UDPGNAc u'
AICAR v'
Inosine w'
R1P x'
IMP y'
AMP z'
ATP aa'
ADPR ab'
Fraction

Figure S5. CA PD-OTC of CZ022 displays enhanced glycolysis, Krebs cycle metabolism, PPP, GNG, riboneogenesis, purine/sugar nucleotide synthesis, and ADP ribosylation.
Freshly prepared CA (■) and NC (●) OTC of CZ022 were cultured and subjected to tracer treatment as in **Fig. 1** (n = 3 biological replicates). The SIRM data were presented as µmole/g residue (**A**) and fraction (**B**). All symbols and abbreviations are as in **Figs. 1-4**. See **Table S5** for statistics.

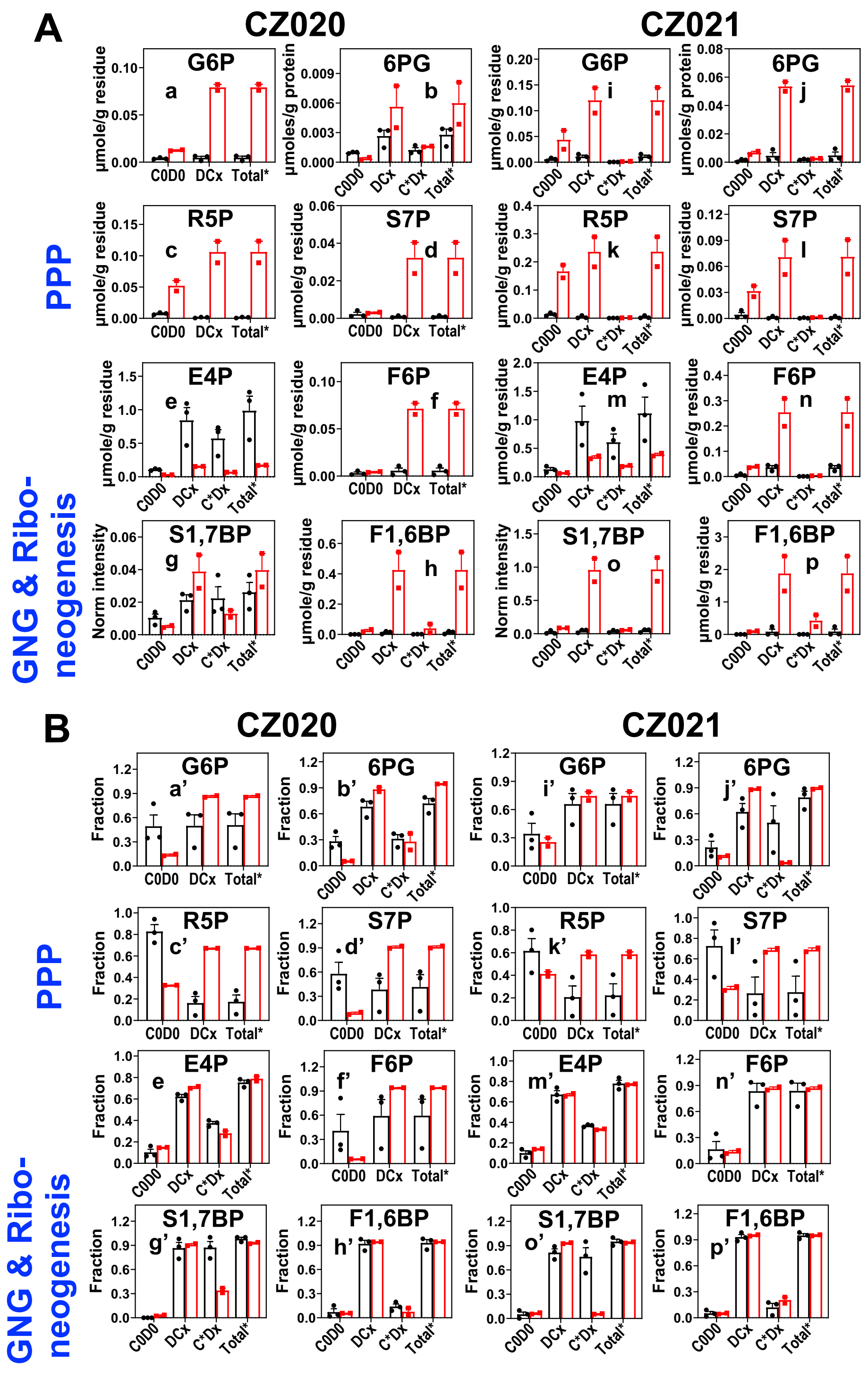

Figure S6. CA PD-OTC of CZ020-021 displays enhanced PPP, GNG, and riboneogenesis.
The SIRM data from **Fig. S2** were analyzed for metabolites of the PPP, GNG, and riboneogenesis pathways as µmole/g residue (**A**) and fraction (**B**). All symbols and abbreviations are as in **Fig. 2**. See **Table S4** for statistics.

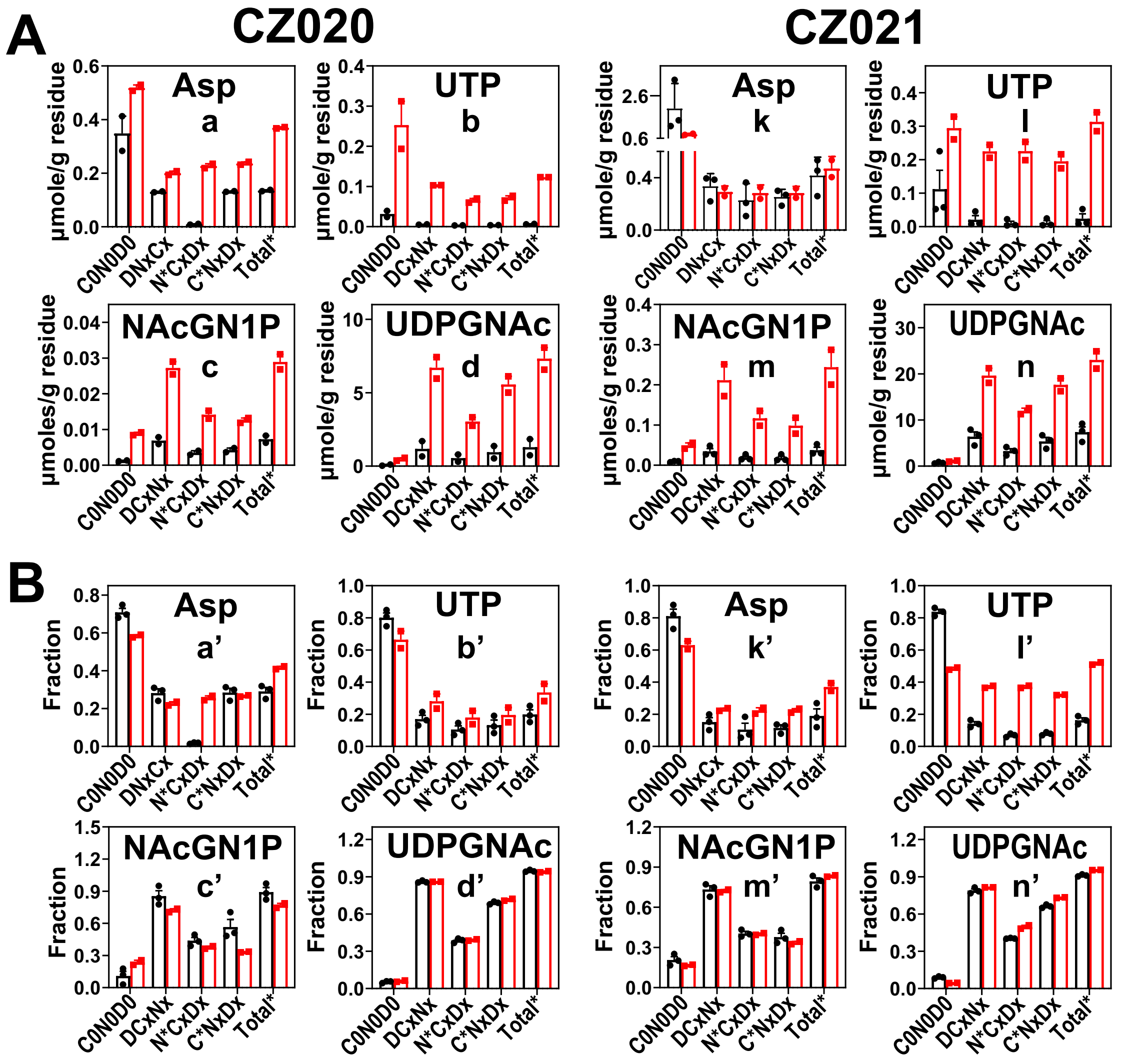

Figure S7. CA PD-OTC of CZ020-021 displays enhanced synthesis of pyrimidine/sugar nucleotides and ADP-ribosylation.

The SIRM data from **Fig. S2** were analyzed for metabolites of the pyrimidine nucleotide/UDP-GlcNAc synthesis pathways as μmole/g residue (**A**) and fraction (**B**). All symbols and abbreviations are as in **Fig. 3**. See **Table S4** for statistics.

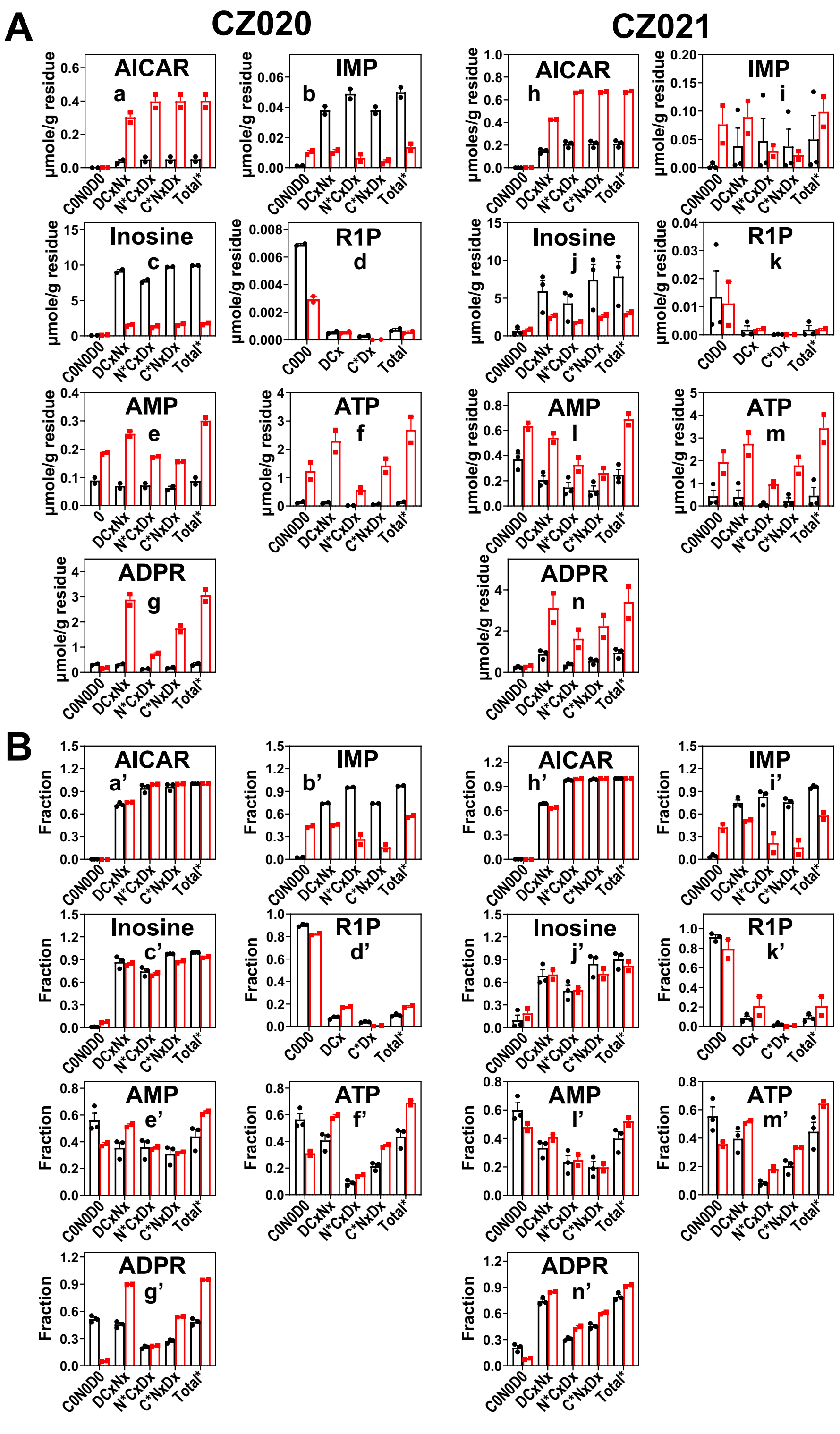

Figure S8. CA PD-OTC of CZ020-021 displays enhanced synthesis of purine nucleotides and ADP-ribosylation.
The SIRM data from **Fig. S2** were analyzed for metabolites of the *de novo*/salvage synthesis pathways of purine nucleotides and ADP-ribose as µmole/g residue (**A**) and fraction (**B**). All symbols and abbreviations are as in **Fig. 4**. See **Table S4** for statistics.

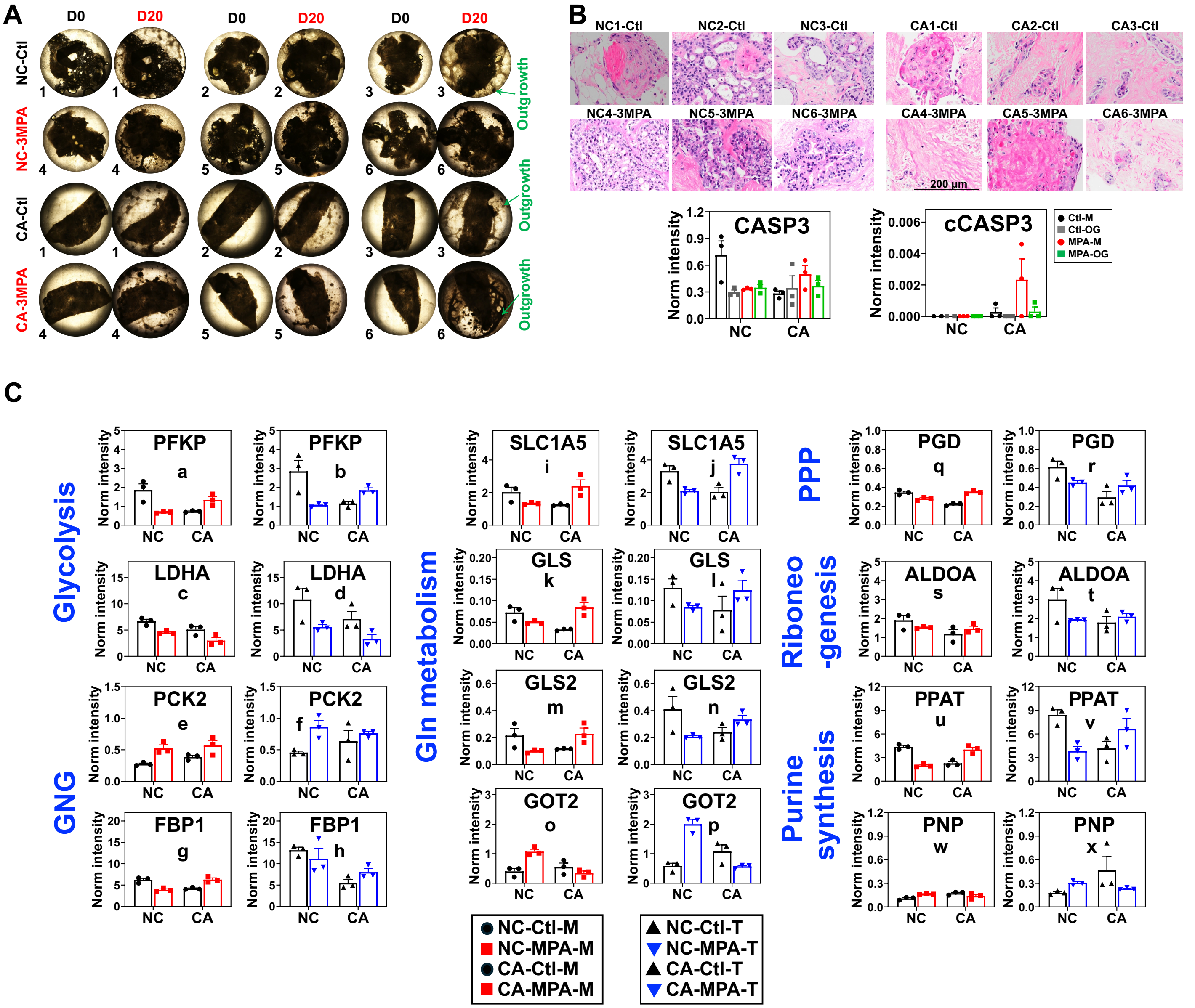

Figure S9. PCK inhibition reduces tissue outgrowth/cellularity and enhances apoptosis in CA but not in NC PD-OTC of CZ017.

Tissue outgrowth (**A**) and H&E-stained images (**B**) of the main tissues from the 3-MPA treatment experiment in **Fig. 6** are shown along with the levels of caspase 3 (CASP3) and cleaved caspase 3 (cCASP3) in the main tissue (**M**) and outgrowth (**OG**) of CA versus NC OTC. Shown in **C** are 3-MPA-induced changes in relevant metabolic proteins that could in part account for the disrupted metabolic network observed for CA versus NC OTC in **Fig. 6**. See **Table S7** for statistics.

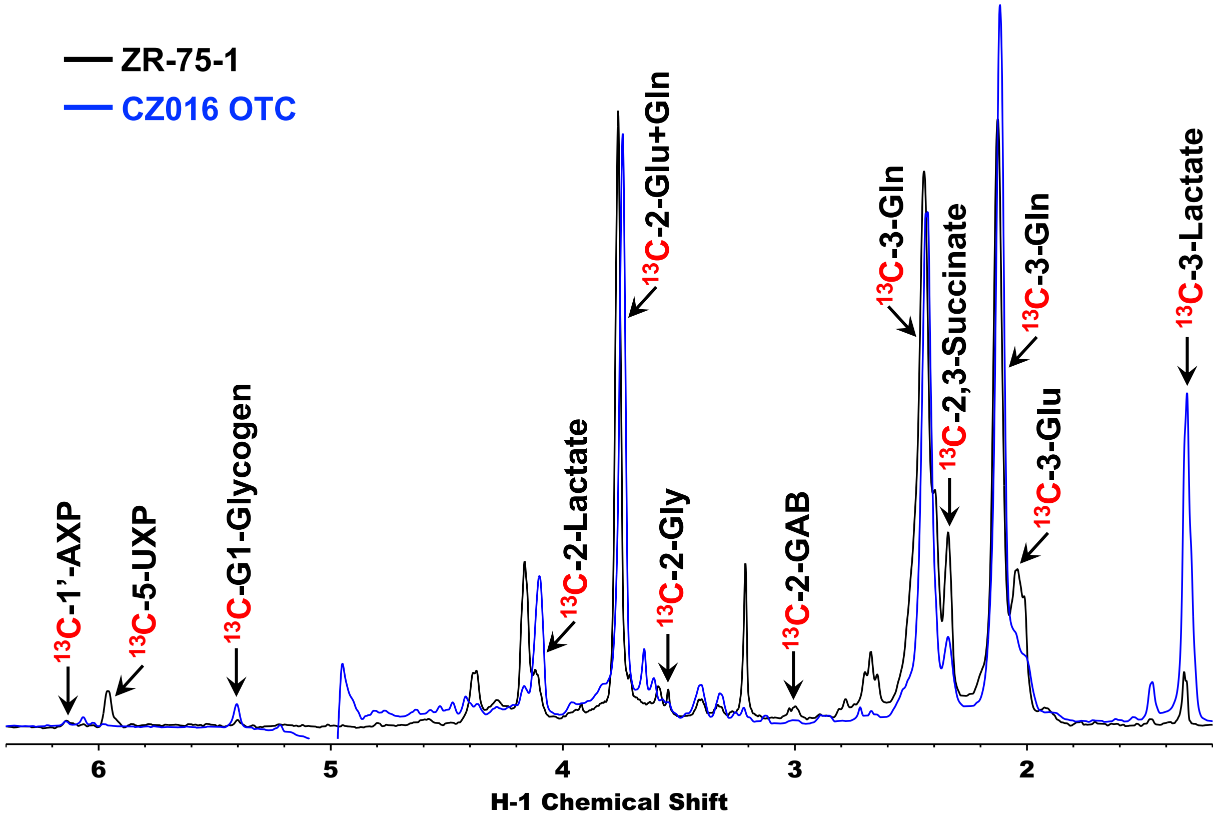

Figure S10. HER2^{+} ZR-75-1 cells and HER2^{-} CA PD-OTC are active in GNG-fueled anabolic metabolism but not HER2^{-} MCF-7 cells.

Our previous study of ZR-75-1 (–) and MCF-7 (data not shown) cells in 2D cultures grown in $^{13}C_5$, $^{15}N_2$-Gln (unpublished results; (14)) was compared with CA OTC of CZ016 (–) for their 1D ^{1}H\{HSQC\} NMR spectra of polar extracts. GNG products, ^{13}C-glycogen, ^{13}C-1'-ribose of adenine nucleotides (AXP), and ^{13}C-Gly were evident in both extracts.

Table S1. Composition of breast cancer organoid medium (BCOM)

	Working Conc.	Unit	Vendor	Catlogue #
Advanced DMEM:F12			Thermofisher	12634-010
HEPES	10	mM	Thermofisher	15630-080
GlutaMAX-I	2	mM	Thermofisher	35050-061
B-27	1	x	Thermofisher	17504-044
RSPO1 Conditioned media	20	%	Prepared as described in [a]	
Noggin	0.1	µg/mL	ATCC	ACS-7105
EGF	0.005	µg/mL	ATCC	ACS-7105
FGF-10	0.02	µg/mL	ATCC	ACS-7105
FGF-7	0.005	µg/mL	ATCC	ACS-7105
A83-01	0.212	µg/mL	ATCC	ACS-7105
SB 202190	0.4	µg/mL	ATCC	ACS-7105
Nicotinamide	1.24	mg/mL	ATCC	ACS-7105
N-Acetyl cysteine	0.204	mg/mL	ATCC	ACS-7105
Heregulin	0.037	µg/mL	ATCC	ACS-7105
Anti anti	1	%	Corning	30004144
Primosin	50	µg/mL	InvivoGen	ant-pm-1
Y27632	5	µM	Selleck Chemicals	101763-964
β-estradiol	10	nM	Sigma Aldrich	E2758

[a] R-Spondin 1 condition medium (RSPO1-CM) used in the OTC of CZ16-17 and CZ19-CZ22 was prepared as described in (57). Briefly, 293T cells (#3710-001-01, Trevigen) were cultured in advanced DMEM/F12 with 10% FBS, 1% Pen Strep and 300 µg/mL zeocin until confluence, and then changed to advanced DMEM/F12 supplemented with 10 mM HEPES, 2mM L-GlutaMAX and 1x Pen Strep before medium collection. RSPO1-CM used in PCKi (3-MPA) experiments with CZ17 OTC was prepared as described per vendor's protocol. Briefly, 293T cells (#SCC111, Sigma-Aldrich) were cultured in advanced DMEM/F12 (12634-010, Gibco) with 10% FBS, 1% Pen Strep until confluence, and then changed to advanced DMEM/F12 supplemented with 10 mM HEPES (15630-060, Gibco), 2 mM L-glutamine (25030-081, Gibco) and 1x Pen Strep (15140-122, Gibco) before medium collection.

Table S2. Primary antibody information

Antigen	Source	Catalogue #	Dilution
ALDOA	Proteintech	11217-1-AP	150
ER	Cell Signaling Tech.	13258	50
FBP1	Proteintech	12842-1-AP	100
GLS	Proteintech	66265-1-Ig	50
GLS2	Invitrogen	PA5-72963	100
GOT2	St John's Laboratory	stj99292	100
GYS1	Proteintech	10566-1-AP	100
Ki67	Proteintech	27309-1-AP	200
LDHA	Proteintech	19987-1-AP	100
PAR	Trevigen	4335-MC-100	500
PARP1	Proteintech	66520-1-Ig	100
PARP9	Proteintech	17535-1-AP	100
PCK2	Proteintech	14892-1-AP	100
PFKP	Proteintech	13389-1-AP	100
PGD	Proteintech	14718-1-AP	100
PNP	Proteintech	18009-1-AP	50
PPAT	Proteintech	15401-1-AP	100
PYGL	Proteintech	15851-1-AP	100
SLC1A5	Proteintech	20350-1-AP	100

Table S3. T-tests for Figs. 1-4

CZ019	TTEST (p-value) µmole/g residue [1]								
Isotopomers	^{12}C	3-D	3-^{13}C	Total*					
Lactate$_{medium}$	1.43E-01	1.63E-04	4.18E-03	2.20E-04					
Isotopologues	C0D0	DCx	C5	C*Dx	Total*				
Pyruvate	2.41E-01	6.95E-02		1.63E-01	5.13E-02				
Lactate	4.97E-01	4.64E-02		4.44E-02	4.61E-02				
G6P	1.30E-02	1.02E-05			1.01E-05				
6PG	4.85E-01	3.70E-03		1.02E-01	3.24E-03				
R5P	4.39E-03	3.03E-03			3.06E-03				
S7P	2.59E-03	3.98E-03			3.88E-03				
E4P	5.33E-01	6.48E-01		5.51E-01	6.18E-01				
F6P	4.21E-04	1.07E-02			1.08E-02				
S1,7BP	3.12E-02	3.85E-02		3.24E-01	4.13E-02				
F1,6BP	4.12E-03	2.42E-02		7.89E-02	2.42E-02				
R1P	9.68E-01	1.15E-01			1.13E-01				
Succinate	2.35E-01	2.78E-06		9.03E-04	7.05E-04				
Fumarate	2.35E-02	1.06E-04		4.27E-03	3.78E-03				
aKG	9.06E-06	2.96E-05	1.45E-03	8.33E-04	6.19E-04				
Isotopologues	C0D0	DCx	C1Dx	C2Dx	C3Dx	C4Dx	C5	C*Dx	Total*
Citrate	1.34E-02	8.94E-03	4.44E-03	9.10E-04	1.65E-04	1.31E-03	6.30E-05	1.16E-03	1.60E-03
Isotopologues	C0D0	DCx	C1Dx	C3	C*Dx	Total*			
Malate	1.61E-02	1.26E-06	5.15E-04	6.01E-03	2.51E-03	2.09E-03			
Isotopologues	C0N0D0	DCx	N*CxDx	C5N1	C*NxDx	Total*			
Glu	7.90E-05	3.39E-03		1.46E-03	2.04E-03	8.20E-04			
GSH	9.09E-03	1.58E-04			4.00E-05	1.92E-05			
Asp	4.48E-01	1.87E-03	2.33E-03		6.50E-03	3.47E-03			
UTP	7.05E-03	8.36E-03	1.82E-02		6.85E-03	1.32E-02			
AICAR	3.74E-01	5.42E-03	7.25E-03		7.09E-03	7.33E-03			
Inosine	4.77E-03	6.54E-01	7.01E-01		5.74E-01	5.75E-01			
IMP	5.11E-02	9.50E-01	8.71E-01		1.88E-01	6.74E-01			
AMP	3.66E-02	5.30E-02	2.29E-01		3.19E-01	6.10E-02			
ATP	3.90E-02	6.23E-02	4.08E-02		5.40E-02	5.66E-02			
ADPR	6.08E-01	1.54E-02	2.59E-02		1.75E-02	1.49E-02			
UDP-GlcNAc	2.67E-02	2.16E-02	2.08E-02		2.08E-02	2.19E-02			
NAcGN1P	8.36E-03	4.24E-03	5.13E-03		5.00E-03	4.44E-03			

[1] Two-tailed unpaired t-test raw p values for µmole/g residue

Table S3 continued

CZ019	*TTEST (p-value) fraction [2]*								
Isotopologues	***^{12}C***	***3-D***	***$3\text{-}^{13}C$***	***Total****					
$Lactate_{medium}$	*1.84E-03*	*1.58E-03*	*5.17E-02*						
Isotopologues	***C0D0***	***DCx***	***C5***	***C*Dx***	***Total****				
Pyruvate	*2.08E-01*	*2.51E-01*		*7.75E-01*	*2.08E-01*				
Lactate	*3.80E-03*	*3.94E-03*		*5.04E-03*	*3.80E-03*				
G6P	*2.06E-01*	*2.07E-01*		*4.14E-01*	*2.06E-01*				
6PG	*7.98E-02*	*1.45E-01*		*7.59E-01*	*7.98E-02*				
R5P	*3.21E-01*	*3.11E-01*		*1.17E-01*	*3.21E-01*				
S7P	*4.90E-01*	*3.27E-01*		*8.47E-02*	*4.90E-01*				
E4P	*6.50E-01*	*3.20E-01*		*9.27E-01*	*5.15E-01*				
F6P	*2.32E-01*	*2.26E-01*		*2.31E-01*	*2.32E-01*				
S1,7BP	*8.22E-03*	*9.89E-01*		*2.83E-01*	*1.41E-03*				
F1,6BP	*8.39E-01*	*7.03E-01*		*9.02E-01*	*8.39E-01*				
R1P	*1.09E-01*	*1.14E-01*		*5.51E-01*	*1.09E-01*				
Succinate	*6.78E-04*	*2.89E-05*		*8.80E-04*	*6.78E-04*				
Fumarate	*2.46E-04*	*2.35E-03*		*2.86E-04*	*2.46E-04*				
αKG	*1.23E-04*	*7.13E-02*	*1.26E-03*	*2.31E-04*	*1.23E-04*				
Isotopologues	***C0D0***	***DCx***	***C1Dx***	***C2Dx***	***C3Dx***	***C4Dx***	***C5***	***C*Dx***	***Total****
Citrate	*2.41E-02*	*1.60E-02*	*8.20E-03*	*3.21E-02*	*6.73E-02*	*5.76E-02*	*2.24E-02*	*2.30E-02*	*2.41E-02*
Isotopologues	***C0D0***	***DCx***	***C1Dx***	***C3***	***C*Dx***	***Total****			
Malate	*1.47E-02*	*1.24E-03*	*7.49E-03*	*1.65E-03*	*1.63E-02*	*1.47E-02*			
Isotopologues	***C0N0D0***	***DCx***	***N*CxDx***	***C5N1***	***C*NxDx***	***Total****			
Glu	*3.75E-02*	*1.41E-02*		*7.50E-01*	*4.24E-02*	*3.75E-02*			
GSH	*1.13E-02*	*9.44E-02*			*3.04E-01*	*1.18E-02*			
Asp	*4.52E-02*	*3.02E-01*	*3.20E-04*		*2.02E-01*	*4.52E-02*			
UTP	*7.70E-04*	*1.85E-03*	*6.86E-03*		*2.28E-02*	*7.70E-04*			
AICAR	*3.74E-01*	*6.81E-01*	*1.55E-01*		*2.63E-01*	*3.74E-01*			
Inosine	*1.33E-02*	*8.92E-01*	*7.55E-01*		*8.18E-02*	*1.33E-02*			
IMP	*3.13E-02*	*8.25E-04*	*6.19E-02*		*5.40E-04*	*3.13E-02*			
AMP	*1.38E-01*	*1.48E-01*	*8.23E-02*		*8.70E-02*	*1.38E-01*			
ATP	*2.72E-01*	*6.33E-01*	*5.00E-01*		*3.89E-01*	*2.72E-01*			
ADPR	*1.52E-02*	*1.29E-02*	*3.36E-01*		*1.98E-02*	*1.52E-02*			
UDP-GlcNAc	*3.50E-01*	*6.57E-01*	*9.94E-01*		*1.28E-01*	*3.50E-01*			
NAcGN1P	*4.35E-01*	*2.37E-01*	*1.07E-01*		*1.81E-01*	*4.35E-01*			

[2] Two-tailed unpaired t-test raw p values for mole fractions

Table S4. T-tests for Figs. S2, S6-S8

CZ020	*TTEST (p-value) µmole/g protein* [1]							
Isotopomers	***^{12}C***	***3-D***	***3-^{13}C***	***Total****				
$Lactate_{medium}$	9.87E-02	9.29E-04	9.37E-03	1.08E-03				
Isotopologues	***C0D0***	***DCx***	***C5***	***C*Dx***	***Total****			
Pyruvate	4.93E-01	6.75E-02		9.40E-02	4.75E-02			
Lactate	6.18E-01	1.03E-04		9.91E-05	9.49E-05			
G6P	1.50E-03	1.66E-04		1.69E-01	1.72E-04			
6PG	7.91E-03	1.89E-01		3.27E-01	1.66E-01			
R5P	5.86E-03	3.92E-03		7.54E-01	3.93E-03			
S7P	5.85E-01	1.49E-02		3.96E-01	1.50E-02			
E4P	5.47E-03	6.86E-02		6.00E-02	6.42E-02			
F6P	4.80E-01	1.43E-03		3.98E-01	1.43E-03			
S1,7BP	1.86E-01	1.39E-01		3.91E-01	3.02E-01			
F1,6BP	2.22E-02	1.87E-02		1.79E-01	1.88E-02			
G1P	4.30E-02	3.17E-03		3.55E-03	3.17E-03			
PRPP	1.27E-02	1.23E-01		7.71E-01	1.66E-01			
R1P	2.40E-01	5.32E-01		9.58E-01	9.58E-01			
αKG	4.89E-02	1.00E-02	5.60E-03	6.73E-03	5.86E-03			
Succinate	2.18E-01	2.03E-07		1.00E-04	3.22E-05			
Fumarate	4.86E-01	6.26E-05		2.28E-04	1.88E-04			
Isotopologues	***C0D0***	***DCx***	***C1Dx***	***C2Dx***	***C3Dx***	***C4Dx***	***C5***	***C*Dx***	***Total****
Citrate	7.84E-03	6.38E-04	5.29E-06	7.50E-05	1.18E-04	4.07E-04	1.25E-04	3.63E-05	6.13E-05
Isotopologues	***C0D0***	***DCx***	***C1Dx***	***C3***	***C*Dx***	***Total****			
Malate	9.00E-01	2.25E-04	4.21E-05	4.11E-04	3.46E-04	2.81E-04			
Isotopologues	***C0N0D0***	***DCx***	***N*CxDx***	***C5N1***	***C*NxDx***	***Total****			
Asp	1.02E-01	5.98E-02	6.56E-05		2.68E-02	4.32E-03			
Glu	1.55E-02	1.67E-04		1.81E-03	1.77E-04	1.42E-04			
GSH	8.30E-01	8.40E-04			8.59E-04	8.25E-04			
UTP	1.54E-02	1.29E-05	5.39E-04		6.07E-04	1.13E-05			
AICAR	1.05E-01	2.14E-03	2.11E-03		2.12E-03	2.09E-03			
Inosine	3.18E-02	3.91E-02	3.49E-02		1.78E-02	1.83E-02			
AMP	1.33E-02	5.23E-03	7.52E-03		5.59E-03	5.76E-03			
IMP	2.05E-02	1.19E-02	9.18E-03		6.39E-03	1.21E-02			
ATP	1.59E-02	4.80E-03	3.69E-03		4.38E-03	4.84E-03			
ADPR	2.27E-01	7.25E-04	2.06E-03		7.15E-04	7.84E-04			
UDP-GlcNAc	1.26E-02	5.00E-03	4.45E-03		4.12E-03	4.53E-03			
NAcGN1P	5.60E-04	2.56E-03	3.36E-03		1.83E-02	2.48E-03			

[1] Two-tailed unpaired t-test raw p values for µmole/g residue

Table S4 continued

CZ020	*TTEST (p-value) fraction* [2]								
Isotopomers	***^{12}C***	***3-D***	***3-^{13}C***	***Total****					
Lactate$_{medium}$	*7.86E-01*	*9.96E-01*	*3.94E-01*						
Isotopologues	***C0D0***	***DCx***	***C5***	***C*Dx***	***Total****				
Pyruvate	*9.23E-03*	*1.75E-02*		*1.04E-01*	*9.23E-03*				
Lactate	*4.60E-05*	*5.50E-05*		*1.05E-03*	*4.60E-05*				
G6P	*1.44E-01*	*1.31E-01*		*6.72E-03*	*1.44E-01*				
6PG	*5.42E-02*	*9.44E-02*		*7.57E-01*	*5.42E-02*				
R5P	*9.00E-03*	*7.48E-03*		*6.44E-02*	*9.00E-03*				
S7P	*7.91E-02*	*6.27E-02*		*1.32E-01*	*8.58E-02*				
E4P	*3.19E-01*	*4.84E-02*		*4.24E-02*	*3.66E-01*				
F6P	*2.78E-01*	*2.75E-01*		*1.03E-01*	*2.78E-01*				
S1,7BP	*8.09E-03*	*6.76E-01*		*1.31E-02*	*1.36E-01*				
F1,6BP	*8.13E-01*	*6.96E-01*		*3.02E-01*	*8.13E-01*				
G1P	*4.08E-04*	*3.70E-04*		*2.74E-01*	*4.08E-04*				
PRPP	*1.84E-03*	*6.76E-02*		*2.68E-01*	*4.14E-01*				
R1P	*6.13E-03*	*3.25E-03*		*6.07E-03*	*6.13E-03*				
αKG	*2.90E-03*	*4.63E-01*	*9.75E-05*	*6.32E-03*	*2.90E-03*				
Succinate	*4.95E-02*	*5.44E-02*		*4.69E-02*	*4.95E-02*				
Fumarate	*2.20E-05*	*1.24E-04*		*1.38E-05*	*2.20E-05*				
Isotopologues	***C0D0***	***DCx***	***C1Dx***	***C2Dx***	***C3Dx***	***C4Dx***	***C5***	***C*Dx***	***Total****
Citrate	*7.76E-04*	*1.19E-03*	*7.87E-05*	*1.28E-03*	*1.30E-03*	*1.34E-02*	*2.87E-03*	*6.68E-04*	*7.76E-04*
Isotopologues	***C0D0***	***DCx***	***C1Dx***	***C3***	***C*Dx***	***Total****			
Malate	*8.55E-06*	*8.07E-05*	*1.56E-05*	*3.45E-06*	*4.74E-06*	*8.55E-06*			
Isotopologues	***C0N0D0***	***DCx***	***N*CxDx***	***C5N1***	***C*NxDx***	***Total****			
Asp	*1.90E-02*	*1.42E-01*	*1.16E-04*		*5.67E-01*	*1.90E-02*			
Glu	*2.35E-02*	*6.21E-01*		*7.94E-01*	*1.14E-01*	*2.35E-02*			
GSH	*8.49E-03*	*9.98E-03*			*9.96E-03*	*8.49E-03*			
UTP	*8.75E-02*	*9.40E-02*	*1.76E-01*		*3.20E-01*	*8.75E-02*			
AICAR	*8.00E-02*	*2.24E-01*	*2.90E-01*		*3.88E-01*	*8.00E-02*			
Inosine	*2.65E-03*	*6.83E-01*	*5.29E-01*		*3.24E-03*	*2.65E-03*			
AMP	*1.40E-02*	*9.61E-03*	*4.41E-02*		*5.52E-02*	*1.40E-02*			
IMP	*9.18E-04*	*2.01E-03*	*9.06E-03*		*3.67E-03*	*9.18E-04*			
ATP	*2.18E-02*	*4.38E-02*	*3.78E-02*		*7.53E-03*	*2.18E-02*			
ADPR	*4.61E-04*	*4.34E-04*	*2.75E-01*		*5.23E-04*	*4.61E-04*			
UDP-GlcNAc	*4.36E-01*	*9.72E-01*	*8.12E-01*		*8.31E-02*	*4.36E-01*			
NAcGN1P	*1.19E-01*	*1.37E-01*	*2.14E-01*		*8.09E-02*	*1.19E-01*			

[2] Two-tailed unpaired t-test raw p values for mole fractions

Table S4 continued

CZ021	*TTEST (p-value) µmole/g protein* [1]								
Isotopomers	***^{12}C***	***D***	***^{13}C***	***Total****					
Lactate$_{medium}$	*5.34E-02*	*2.65E-04*	*2.36E-02*	*2.44E-04*					
Isotopologues	***C0D0***	***DCx***	***C5***	***C*Dx***	***Total****				
Pyruvate	*3.30E-01*	*1.96E-01*		*7.75E-01*	*2.35E-01*				
Lactate	*5.04E-01*	*3.39E-03*		*1.28E-03*	*3.28E-03*				
G6P	*7.10E-02*	*1.00E-02*		*1.31E-02*	*1.01E-02*				
6PG	*1.52E-02*	*1.11E-03*		*1.09E-01*	*1.19E-03*				
R5P	*3.22E-03*	*9.99E-03*		*1.60E-02*	*1.00E-02*				
S7P	*1.75E-02*	*1.82E-02*		*1.13E-01*	*1.80E-02*				
E4P	*2.23E-01*	*1.55E-01*		*1.16E-01*	*1.45E-01*				
F6P	*4.12E-03*	*1.39E-02*		*2.51E-03*	*1.38E-02*				
S1,7BP	*3.88E-02*	*6.19E-03*		*2.19E-01*	*6.37E-03*				
PEP	*1.47E-02*	*2.54E-02*		*8.29E-01*	*2.59E-02*				
F1,6BP	*4.16E-03*	*2.26E-02*		*5.25E-02*	*2.26E-02*				
G1P	*4.81E-02*	*2.15E-03*		*7.88E-01*	*2.22E-03*				
PRPP	*1.07E-01*	*5.29E-02*		*8.34E-02*	*6.91E-02*				
R1P	*8.75E-01*	*9.84E-01*		*2.12E-01*	*9.97E-01*				
aKG	*2.88E-01*	*1.23E-01*	*1.71E-02*	*9.17E-03*	*9.79E-03*				
Succinate	*3.45E-01*	*3.01E-02*		*1.54E-02*	*1.70E-02*				
Fumarate	*1.37E-01*	*3.26E-02*		*6.56E-03*	*7.64E-03*				
Isotopologues	***C0D0***	***DCx***	***C1Dx***	***C2Dx***	***C3Dx***	***C4Dx***	***C5***	***C*Dx***	***Total****
Citrate	*9.50E-01*	*1.40E-03*	*1.71E-03*	*3.12E-03*	*4.26E-03*	*1.30E-01*	*6.68E-03*	*3.51E-03*	*3.33E-03*
Isotopologues	***C0D0***	***DCx***	***C1Dx***	***C3***	***C*Dx***	***Total****			
Malate	*3.96E-01*	*6.04E-03*	*3.41E-03*	*9.40E-03*	*4.02E-03*	*2.85E-03*			
Isotopologues	***C0N0D0***	***DCx***	***N*CxDx***	***C5N1***	***C*NxDx***	***Total****			
Asp	*2.68E-01*	*6.26E-01*	*6.16E-01*		*5.89E-01*	*6.71E-01*			
Glu	*1.56E-01*	*4.71E-02*		*8.63E-02*	*9.47E-03*	*1.22E-02*			
GSH	*9.27E-02*	*5.61E-02*			*6.75E-02*	*8.05E-02*			
UTP	*9.96E-02*	*2.47E-03*	*2.18E-03*		*1.94E-03*	*1.89E-03*			
AICAR	*4.95E-01*	*3.68E-04*	*2.59E-04*		*2.95E-04*	*2.82E-04*			
Inosine	*8.59E-01*	*1.72E-01*	*2.11E-01*		*1.64E-01*	*1.53E-01*			
IMP	*6.11E-02*	*3.52E-01*	*7.69E-01*		*7.30E-01*	*4.61E-01*			
AMP	*2.18E-02*	*6.39E-03*	*7.39E-02*		*8.57E-02*	*6.65E-03*			
ATP	*5.99E-02*	*2.31E-02*	*3.59E-03*		*1.92E-02*	*1.96E-02*			
ADPR	*3.57E-01*	*2.72E-02*	*3.32E-02*		*2.70E-02*	*2.72E-02*			
UDP-GlcNAc	*9.63E-02*	*4.93E-03*	*1.83E-03*		*3.88E-03*	*4.55E-03*			
NAcGN1P	*5.23E-03*	*1.03E-02*	*6.78E-03*		*1.48E-02*	*8.77E-03*			

[1] Two-tailed unpaired t-test raw p values for µmole/g residue

Table S4 continued

CZ021	TTEST (p-value) fraction [2]								
Isotopologues	***^{12}C***	***D***	***^{13}C***	***Total****					
$Lactate_{medium}$	*1.90E-01*	*1.20E-01*	*6.91E-01*						
Isotopologues	***C0D0***	***DCx***	***C5***	***C*Dx***	***Total****				
Pyruvate	*5.29E-02*	*6.15E-02*		*1.59E-01*	*5.29E-02*				
Lactate	*1.94E-02*	*2.01E-02*		*1.02E-03*	*1.94E-02*				
G6P	*5.99E-01*	*6.01E-01*		*7.05E-02*	*5.99E-01*				
6PG	*3.46E-01*	*1.30E-01*		*1.63E-01*	*3.46E-01*				
R5P	*2.48E-01*	*6.12E-02*		*9.38E-02*	*7.37E-02*				
S7P	*1.35E-01*	*1.35E-01*		*1.54E-01*	*1.35E-01*				
E4P	*2.76E-01*	*9.63E-01*		*2.68E-02*	*8.77E-01*				
F6P	*8.12E-01*	*8.02E-01*		*2.83E-01*	*8.12E-01*				
S1,7BP	*7.80E-01*	*1.47E-01*		*1.68E-02*	*7.80E-01*				
PEP	*3.52E-02*	*2.10E-01*		*2.07E-02*	*3.52E-02*				
F1,6BP	*9.50E-01*	*6.39E-01*		*2.87E-01*	*9.50E-01*				
G1P	*1.13E-02*	*1.06E-02*		*4.82E-01*	*1.13E-02*				
PRPP	*6.67E-01*	*2.62E-01*		*4.63E-02*	*6.67E-01*				
R1P	*2.24E-01*	*2.14E-01*		*3.18E-01*	*2.24E-01*				
αKG	*1.16E-02*	*9.02E-01*	*8.60E-02*	*1.82E-02*	*1.16E-02*				
Succinate	*1.28E-01*	*1.24E-01*		*1.28E-01*	*1.28E-01*				
Fumarate	*2.24E-02*	*1.07E-01*		*1.94E-02*	*2.24E-02*				
Isotopologues	***C0D0***	***DCx***	***C1Dx***	***C2Dx***	***C3Dx***	***C4Dx***	***C5***	***C*Dx***	***Total****
Citrate	*1.26E-02*	*1.69E-03*	*2.63E-03*	*1.72E-02*	*3.72E-03*	*5.67E-01*	*4.05E-03*	*1.23E-02*	*1.26E-02*
Isotopologues	*C0D0*	*DCx*	*C1Dx*	*C3*	*C*Dx*	*Total**			
Malate	*1.89E-02*	*1.93E-01*	*8.60E-03*	*5.79E-03*	*1.53E-02*	*1.89E-02*			
Isotopologues	***C0N0D0***	***DCx***	***N*CxDx***	***C5N1***	***C*NxDx***	***Total****			
Asp	*5.54E-02*	*1.24E-01*	*1.15E-01*		*2.27E-02*	*5.54E-02*			
Glu	*2.93E-02*	*1.48E-01*		*5.53E-01*	*2.26E-02*	*2.93E-02*			
GSH	*6.53E-02*	*4.35E-02*			*5.82E-02*	*6.53E-02*			
UTP	*3.68E-04*	*8.82E-04*	*7.09E-05*		*6.05E-05*	*3.68E-04*			
AICAR	*4.95E-01*	*4.21E-03*	*9.74E-02*		*2.24E-01*	*4.95E-01*			
Inosine	*4.35E-01*	*9.06E-01*	*9.38E-01*		*3.77E-01*	*4.35E-01*			
IMP	*2.06E-03*	*1.73E-02*	*1.51E-02*		*5.67E-03*	*2.06E-03*			
AMP	*1.68E-01*	*2.15E-01*	*8.41E-01*		*9.83E-01*	*1.68E-01*			
ATP	*1.06E-01*	*1.73E-01*	*9.52E-03*		*4.19E-02*	*1.06E-01*			
ADPR	*3.17E-02*	*3.88E-02*	*1.04E-02*		*9.74E-03*	*3.17E-02*			
UDP-GlcNAc	*5.34E-03*	*2.41E-01*	*3.23E-03*		*6.85E-03*	*5.34E-03*			
NAcGN1P	*3.00E-01*	*7.80E-01*	*8.63E-01*		*3.34E-01*	*3.00E-01*			

[2] Two-tailed unpaired t-test raw p values for mole fractions

Table S5. T-tests for Figs. S4, S5

CZ017	*TTEST (p-value) µmole/g protein* [1]								
Isotopomers	***^{12}C***	***D***	***^{13}C***	***Total****					
$Lactate_{medium}$	*8.39E-02*	*4.66E-02*	*1.09E-02*	*4.37E-02*					
Isotopologues	***C0N0***	***DCx***	***C5***	***C*Dx***	***Total****				
Pyruvate	*7.19E-01*	*2.89E-01*		*2.33E-01*	*2.42E-01*				
Lactate	*8.97E-01*	*3.21E-02*		*2.93E-02*	*3.25E-02*				
Succinate	*8.67E-01*	*3.04E-02*		*1.90E-03*	*1.98E-03*				
G6P	*5.36E-02*	*1.22E-01*		*1.23E-01*	*1.22E-01*				
6PG	*3.95E-01*	*9.74E-01*		*9.67E-01*	*9.79E-01*				
R5P	*3.24E-01*	*3.02E-01*		*3.47E-01*	*3.02E-01*				
S7P	*3.41E-01*	*1.00E-01*		*1.00E-01*	*1.34E-01*				
E4P	*8.59E-01*	*8.06E-01*		*6.27E-01*	*6.22E-01*				
F6P	*3.23E-01*	*6.36E-01*		*9.08E-01*	*6.38E-01*				
S1,7BP	*8.59E-02*	*5.10E-01*		*4.99E-01*	*4.37E-01*				
PEP	*9.50E-01*	*6.96E-01*		*7.04E-01*	*6.90E-01*				
F1,6BP	*7.69E-01*	*5.35E-01*		*4.58E-01*	*5.35E-01*				
G1P	*1.93E-01*	*8.31E-02*		*4.12E-02*	*8.41E-02*				
PRPP	*5.85E-01*	*9.74E-01*		*8.76E-01*	*9.56E-01*				
R1P	*1.45E-01*	*6.84E-01*		*8.33E-01*	*7.42E-01*				
aKG	*8.44E-01*	*9.05E-01*	*1.99E-01*	*2.83E-01*	*2.86E-01*				
Succinate	*8.67E-01*	*3.04E-02*		*1.90E-03*	*1.98E-03*				
Fumarate	*5.92E-01*	*1.14E-01*		*1.34E-01*	*1.46E-01*				
Isotopologues	***C0D0***	***DCx***	***C1Dx***	***C2Dx***	***C3Dx***	***C4Dx***	***C5***	***C*Dx***	***Total****
Citrate	*5.04E-01*	*3.04E-01*	*8.70E-01*	*7.93E-01*	*4.44E-01*	*3.23E-01*	*3.49E-01*	*5.05E-01*	*5.23E-01*
Isotopologues	*C0D0*	*DCx*	*C1Dx*	*C3*	*C*Dx*	*Total**			
Malate	*5.92E-01*	*8.02E-01*	*5.52E-01*		*9.08E-01*	*9.09E-01*			
Isotopologues	***C0N0D0***	***DCxNx***	***N*CxDx***	***C5N1***	***C*NxDx***	***Total****			
Asp	*1.57E-01*	*4.49E-01*	*7.98E-01*		*9.04E-01*	*6.50E-01*			
Glu	*1.89E-01*	*7.86E-01*	*6.73E-01*	*2.93E-01*	*6.47E-01*	*7.98E-01*			
GSH	*1.32E-01*	*4.07E-01*	*4.62E-01*		*4.81E-01*	*6.80E-01*			
UTP	*8.36E-01*	*3.45E-01*	*3.25E-01*		*2.82E-01*	*3.58E-01*			
AICAR	*3.95E-02*	*5.85E-01*	*5.91E-01*		*5.90E-01*	*5.89E-01*			
Inosine	*3.41E-02*	*2.21E-01*	*2.46E-01*		*2.41E-01*	*2.16E-01*			
IMP	*2.52E-01*	*7.17E-01*	*8.46E-01*		*7.97E-01*	*7.19E-01*			
AMP	*3.40E-01*	*9.02E-01*	*4.78E-01*		*7.61E-01*	*8.58E-01*			
ATP	*9.92E-01*	*2.63E-01*	*1.78E-01*		*2.78E-01*	*2.63E-01*			
ADPR	*6.07E-02*	*4.61E-01*	*8.05E-01*		*4.13E-01*	*5.06E-01*			
UDP-GlcNAc	*2.02E-01*	*3.17E-01*	*2.59E-01*		*3.37E-01*	*3.08E-01*			
NAcGN1P	*1.99E-01*	*3.83E-01*	*4.20E-01*		*3.96E-01*	*4.26E-01*			

[1] Two-tailed unpaired t-test raw p values for µmole/g residue

Table S5 continued

CZ017	TTEST (p-value) fraction [2]								
Isotopomers	^{12}C	D	^{13}C	Total*					
Lactate$_{medium}$	8.18E-01	8.78E-01	6.95E-03	8.18E-01					
Isotopologues	C0N0	DCx	C5	C*Dx	Total*				
Pyruvate	1.73E-01	2.37E-01		1.69E-01	1.73E-01				
Lactate	3.14E-02	3.14E-02		2.87E-02	3.14E-02				
Succinate	5.28E-03	2.43E-02		4.68E-03	5.28E-03				
G6P	3.78E-01	3.52E-01		6.78E-01	3.78E-01				
6PG	8.68E-01	9.66E-01		6.87E-01	8.68E-01				
R5P	9.45E-01	9.62E-01		9.75E-01	9.45E-01				
S7P	5.09E-01	8.11E-01		3.21E-01	7.04E-01				
E4P	3.87E-01	8.21E-01		5.23E-01	4.15E-01				
F6P	9.27E-01	9.80E-01		5.89E-02	9.27E-01				
S1,7BP	1.48E-01	4.00E-01		6.57E-02	1.48E-01				
PEP	4.13E-01	4.31E-01		6.50E-01	4.13E-01				
F1,6BP	1.26E-02	1.89E-02		7.24E-02	1.26E-02				
G1P	3.04E-01	3.02E-01		5.53E-01	3.04E-01				
PRPP	7.33E-01	7.54E-01		2.43E-01	7.33E-01				
R1P	2.24E-01	1.96E-01		2.92E-01	2.24E-01				
aKG	7.61E-02	1.08E-01	1.40E-02	7.43E-02	7.61E-02				
Succinate	5.28E-03	2.43E-02		4.68E-03	5.28E-03				
Fumarate	4.56E-01	4.39E-01		4.49E-01	4.56E-01				
Isotopologues	C0D0	DCx	C1Dx	C2Dx	C3Dx	C4Dx	C5	C*Dx	Total*
Citrate	2.34E-02	4.18E-01	6.96E-01	3.30E-01	3.33E-01	3.80E-01	3.44E-01	3.27E-01	3.37E-01
Isotopologues	C0D0	DCx	C1Dx	C3	C*Dx	Total*			
Malate	6.95E-01	6.38E-01	5.48E-01		6.94E-01	6.95E-01			
Isotopologues	C0N0D0	DCx	N*CxDx	C5N1	C*NxDx	Total*			
Asp	1.31E-01	6.41E-01	5.56E-02		6.22E-02	1.31E-01			
Glu	4.78E-02	7.15E-03	1.09E-01	3.21E-01	4.48E-02	4.78E-02			
GSH	1.87E-01	1.25E-01	1.26E-01		1.27E-01	1.87E-01			
UTP	8.96E-03	5.59E-03	3.58E-03		7.47E-03	8.96E-03			
AICAR	6.22E-03	7.50E-01	7.95E-01		7.68E-01	6.22E-03			
Inosine	9.86E-01	7.16E-01	8.79E-01		6.83E-01	9.86E-01			
IMP	1.86E-02	9.53E-03	4.10E-02		5.86E-02	1.86E-02			
AMP	5.70E-03	1.47E-02	6.04E-02		5.69E-02	5.70E-03			
ATP	1.43E-03	7.21E-04	3.05E-02		8.73E-03	1.43E-03			
ADPR	7.59E-02	5.20E-02	4.43E-01		4.30E-02	7.59E-02			
UDP-GlcNAc	5.01E-02	4.93E-02	5.47E-02		2.73E-02	5.01E-02			
NAcGN1P	4.10E-01	3.13E-01	3.26E-01		3.10E-01	3.32E-01			

[2] Two-tailed unpaired t-test raw p values for mole fractions

Table S5 continued

CZ022	*TTEST (p-value) μmole/g protein* [1]								
Isotopomers	***^{12}C***	***D***	***^{13}C***	***Total****					
$Lactate_{medium}$	*7.95E-02*	*3.82E-02*	*9.83E-02*	*4.03E-02*					
Isotopologues	*C0D0*	*DCx*	*C5*	*C*Dx*	*Total**				
Pyruvate	*8.64E-01*	*1.48E-01*		*1.58E-01*	*1.57E-01*				
Lactate	*4.33E-02*	*1.54E-03*		*9.49E-03*	*1.61E-03*				
G6P	*2.04E-02*	*7.71E-02*		*3.53E-01*	*7.71E-02*				
6PG	*5.06E-02*	*4.30E-03*		*5.48E-01*	*3.72E-03*				
R5P	*3.46E-03*	*1.05E-02*		*1.36E-01*	*1.06E-02*				
S7P	*1.58E-01*	*4.57E-02*		*4.31E-01*	*4.58E-02*				
E4P	*7.04E-01*	*4.45E-01*		*2.61E-01*	*3.75E-01*				
F6P	*3.97E-02*	*8.27E-02*		*5.56E-01*	*8.28E-02*				
S1,7BP	*1.58E-01*	*4.57E-02*		*4.31E-01*	*4.58E-02*				
PEP	*5.60E-03*	*2.21E-03*		*3.43E-02*	*2.20E-03*				
F1,6BP	*2.74E-02*	*7.78E-03*		*1.06E-02*	*8.00E-03*				
G1P	*7.94E-03*	*7.08E-03*		*1.93E-01*	*7.03E-03*				
PRPP	*3.39E-01*	*3.72E-01*		*4.49E-01*	*3.74E-01*				
R1P	*8.99E-01*	*1.08E-02*		*4.30E-01*	*1.99E-02*				
αKG	*5.79E-02*	*6.67E-02*	*1.91E-02*	*3.30E-02*	*2.80E-02*				
Succinate	*7.95E-01*	*9.85E-03*		*6.39E-03*	*4.34E-03*				
Fumarate	*1.93E-01*	*9.45E-02*		*3.16E-02*	*3.35E-02*				
Isotopologues	***0***	***DCx***	***C1Dx***	***C2Dx***	***C3Dx***	***C4Dx***	***C5***	***C*Dx***	***Total****
Citrate	*1.28E-02*	*5.43E-03*	*4.14E-03*	*1.29E-02*	*2.79E-02*	*4.32E-03*	*1.48E-02*	*6.22E-03*	*5.03E-03*
Isotopologues	***C0D0***	***DCx***	***C1Dx***	***C3***	***C*Dx***	***Total****			
Malate	*3.93E-02*	*2.82E-02*	*3.77E-02*		*2.38E-02*	*2.23E-02*			
Isotopologues	***C0N0D0***	***DCx***	***N*CxDx***	***C5N1***	***C*NxDx***	***Total****			
Asp	*2.55E-01*	*3.58E-01*	*4.77E-01*		*5.51E-01*	*4.55E-01*			
Glu	*2.67E-01*	*1.85E-03*	*2.36E-02*	*1.91E-02*	*8.21E-03*	*1.10E-02*			
GSH	*7.02E-01*	*1.61E-01*		*9.00E-02*	*1.59E-01*	*1.75E-01*			
UTP	*9.45E-01*	*5.84E-01*	*5.68E-01*		*4.76E-01*	*6.15E-01*			
AICAR	*6.20E-01*	*1.66E-02*	*1.30E-02*		*1.31E-02*	*1.32E-02*			
Inosine	*5.20E-02*	*3.55E-01*	*2.56E-01*		*3.06E-01*	*3.33E-01*			
IMP	*4.46E-02*	*6.85E-02*	*1.06E-02*		*3.09E-02*	*5.42E-02*			
AMP	*1.09E-02*	*4.01E-02*	*7.44E-02*		*1.13E-01*	*4.01E-02*			
ATP	*4.53E-01*	*3.82E-01*	*6.22E-01*		*4.15E-01*	*4.33E-01*			
ADPR	*5.58E-01*	*1.24E-01*	*1.01E-01*		*1.14E-01*	*1.28E-01*			
UDP-GlcNAc	*3.81E-01*	*7.14E-02*	*6.86E-02*		*7.12E-02*	*7.52E-02*			
NAcGN1P	*1.08E-02*	*3.69E-03*	*2.99E-03*		*6.29E-03*	*3.71E-03*			

[1] Two-tailed unpaired t-test raw p values for μmole/g residue

Table S5 continued

CZ022	TTEST (p-value) fraction [2]								
**Isotopomers**	**^{12}C**	**D**	**^{13}C**	**Total***					
Lactate$_{medium}$	3.99E-03	5.19E-03	2.62E-01	3.99E-03					
**Isotopologues**	**C0D0**	**DCx**	**C5**	**C*Dx**	**Total***				
Pyruvate	1.84E-02	1.64E-02		3.05E-02	1.84E-02				
Lactate	1.63E-02	1.62E-02		1.24E-02	1.63E-02				
G6P	1.36E-01	1.35E-01		3.77E-01	1.36E-01				
6PG	1.32E-01	1.49E-01		1.71E-01	1.32E-01				
R5P	2.31E-02	2.35E-02		1.81E-01	2.31E-02				
S7P	3.08E-01	2.66E-01		3.04E-01	3.08E-01				
E4P	8.82E-01	5.38E-01		5.58E-01	7.48E-01				
F6P	1.71E-01	1.67E-01		3.70E-01	1.71E-01				
S1,7BP	4.09E-02	9.08E-01		5.57E-03	4.09E-02				
PEP	6.83E-02	9.78E-02		2.30E-01	6.83E-02				
F1,6BP	2.61E-01	3.64E-01		7.24E-03	2.61E-01				
G1P	7.09E-01	7.11E-01		4.45E-01	7.09E-01				
PRPP	1.26E-01	3.24E-01		2.13E-01	1.26E-01				
R1P	7.75E-02	7.92E-02		7.42E-02	7.75E-02				
αKG	7.67E-02	5.51E-01	1.56E-01	1.18E-01	7.67E-02				
Succinate	1.12E-02	1.08E-01		8.38E-03	1.12E-02				
Fumarate	2.22E-02	1.04E-01		1.89E-02	2.22E-02				
**Isotopologues**	**0**	**DCx**	**C1Dx**	**C2Dx**	**C3Dx**	**C4Dx**	**C5**	**C*Dx**	**Total***
Citrate	7.17E-02	1.46E-02	9.51E-03	2.06E-01	9.40E-01	5.83E-02	9.28E-01	1.01E-01	7.17E-02
**Isotopologues**	**C0D0**	**DCx**	**C1Dx**	**C3**	**C*Dx**	**Total***			
Malate	9.71E-02	3.38E-02	1.51E-01		1.17E-01	9.71E-02			
**Isotopologues**	**C0N0D0**	**DCx**	**N*CxDx**	**C5N1**	**C*NxDx**	**Total***			
Asp	9.57E-01	8.84E-01	7.34E-01		6.74E-01	9.57E-01			
Glu	1.57E-01	7.48E-01	2.50E-01	5.18E-01	4.95E-01	1.57E-01			
GSH	9.54E-02	1.23E-01		1.73E-01	1.61E-01	9.54E-02			
UTP	5.00E-01	3.73E-01	7.60E-01		6.51E-01	5.00E-01			
AICAR	3.68E-01	9.16E-02	1.72E-01		8.87E-02	3.68E-01			
Inosine	8.82E-04	5.60E-01	2.63E-02		6.31E-04	8.82E-04			
IMP	1.74E-02	1.49E-02	7.82E-03		1.11E-02	1.74E-02			
AMP	4.09E-01	3.02E-01	8.28E-02		2.45E-01	4.09E-01			
ATP	2.04E-01	1.29E-01	4.72E-01		1.53E-01	2.04E-01			
ADPR	2.28E-01	1.73E-01	2.32E-02		8.40E-02	2.28E-01			
UDP-GlcNAc	1.01E-03	1.03E-02	6.06E-01		3.80E-03	1.01E-03			
NAcGN1P	3.50E-01	3.87E-01	4.76E-01		2.16E-01	3.50E-01			

[2] Two-tailed unpaired t-test raw p values for mole fractions

Table S6. T-tests for Fig. 5

	TTEST (p-value) Normalized intensity [1]		
Protein targets	***CZ019***	***CZ020***	***CZ021***
PKM2	*1.24E-02*	*1.25E-01*	*1.66E-01*
HK2	*1.08E-01*	*1.63E-01*	*1.77E-01*
LDHA	*1.01E-02*	*1.51E-01*	*2.37E-01*
CS	*1.12E-02*	*1.31E-01*	*1.49E-01*
OGDH	*6.14E-02*	*1.50E-01*	*2.48E-01*
MDH2	*1.43E-02*	*1.20E-01*	*1.44E-01*
SDHA	*2.66E-02*	*1.92E-01*	*2.00E-01*
ME1	*3.46E-02*	*1.79E-01*	*2.45E-01*
ACLY	*1.05E-02*	*1.59E-01*	*2.29E-01*
MDH1	*1.78E-02*	*1.67E-01*	*2.44E-01*
GLS	*1.40E-01*	*1.56E-01*	*2.54E-01*
GOT2	*4.69E-01*	*1.99E-01*	*4.82E-01*
TKT	*1.87E-02*	*1.60E-01*	*3.69E-01*
ALDOA	*9.88E-03*	*1.27E-01*	*1.84E-01*
PCK2	*2.84E-01*	*2.03E-01*	*1.95E-01*
CAD	*2.01E-01*	*1.60E-01*	*2.12E-01*
DHODH	*3.22E-01*	*1.81E-01*	*3.03E-01*
PPAT	*2.09E-01*	*9.85E-02*	*2.07E-01*
PNP	*2.65E-01*	*2.19E-01*	*4.39E-01*
PAR	*1.77E-02*	*1.47E-01*	*2.14E-01*
PARP1	*5.74E-03*	*1.55E-01*	*1.91E-01*
GlcNAc	*1.40E-01*	*1.19E-01*	*1.62E-01*

[1] Two-tailed unpaired t-test raw p values

Table S7. T-tests for Fig. 9A-C

NC ctl-M vs MPA-M (for Fig. 9A)

CZ017	TTEST (p-value) µmole/g residue				
**Isotopologues**	**C0D0**	**DCx**	**C*Dx**	**Total***	
1,3BPG	9.13E-02	4.11E-02		4.11E-02	
S1,7BP	1.85E-01	1.63E-01		1.46E-01	
F1,6BP	4.47E-01	7.94E-01	5.44E-01	7.93E-01	
R1P	9.73E-02	6.08E-01		6.11E-01	
PRPP	4.60E-02	6.89E-02		6.65E-02	
**Isotopologues**	**C0N0D0**	**DCxNx**	**N*CxDx**	**C*NxDx**	**Total***
GSH	2.57E-02	1.10E-02		3.40E-03	2.04E-03
IMP	6.67E-04	5.40E-02	6.07E-01	9.63E-01	4.65E-02
ATP	5.97E-03	2.09E-01	2.74E-01	3.85E-01	2.15E-01
ADPR	1.05E-02	5.87E-03	1.98E-02	3.28E-02	4.66E-03

CA ctl-M vs MPA-M (for Fig. 9A)

CZ017	TTEST (p-value) µmole/g residue				
**Isotopologues**	**C0D0**	**DCx**	**C*Dx**	**Total***	
1,3BPG	8.77E-03	6.95E-02		6.91E-02	
S1,7BP	1.18E-01	9.20E-01		3.64E-01	
F1,6BP	1.01E-02	5.32E-02	3.43E-02	5.32E-02	
PRPP	8.95E-03	5.21E-02		5.33E-02	
R1P	9.45E-03	4.28E-05		4.56E-05	
**Isotopologues**	**C0N0D0**	**DCxNx**	**N*CxDx**	**C*NxDx**	**Total***
GSH	7.59E-03	2.64E-02		2.66E-02	3.11E-02
Inosine	4.03E-01	9.83E-01	8.63E-01	7.50E-01	9.61E-01
IMP	6.82E-03	8.15E-02	3.15E-02	1.01E-01	3.61E-02
ATP	7.86E-03	1.41E-01	6.34E-01	4.82E-01	1.52E-01
ADPR	1.69E-01	2.92E-02	1.20E-02	1.35E-02	2.92E-02

NC ctl-T vs MPA-T T: main tissue + outgrowth (for Fig. 9A)

CZ017	TTEST (p-value) µmole/g residue				
**Isotopologues**	**C0D0**	**DCx**	**C*Dx**	**Total***	
1,3BPG	7.10E-02	2.76E-02		2.74E-02	
S1,7BP	4.31E-01	5.86E-04		1.72E-03	
F1,6BP	2.25E-03	6.04E-01	2.99E-01	6.02E-01	
PRPP	3.09E-02	8.70E-02		8.33E-02	
R1P	3.14E-02	2.54E-01		2.63E-01	
**Isotopologues**	**C0N0D0**	**DCxNx**	**N*CxDx**	**C*NxDx**	**Total***
GSH	5.63E-02	4.78E-02		1.09E-02	3.29E-03
Inosine	2.95E-02	4.51E-02	4.48E-02	4.30E-02	4.22E-02

IMP	6.51E-03	4.39E-02	5.99E-01	5.06E-01	3.24E-02
ATP	4.21E-03	1.73E-01	2.28E-01	3.64E-01	1.79E-01
ADPR	9.01E-01	1.04E-02	1.77E-02	1.00E-02	8.28E-03

CA ctl-T vs MPA-T   T: main tissue + outgrowth (for Fig. 9A)

**CZ017**	**TTEST (p-value) µmole/g residue**				
**Isotopologues**	**C0D0**	**DCx**	**C*Dx**	**Total***	
1,3BPG	1.07E-01	3.17E-02		3.16E-02	
S1,7BP	4.61E-01	5.30E-01		5.71E-01	
F1,6BP	2.65E-01	4.89E-02	2.74E-01	5.35E-02	
PRPP	2.45E-01	1.31E-02		2.33E-02	
R1P	1.21E-01	9.66E-02		9.70E-02	
**Isotopologues**	**C0N0D0**	**DCxNx**	**N*CxDx**	**C*NxDx**	**Total***
GSH	2.06E-03	2.63E-01		2.21E-01	1.71E-01
Inosine	2.02E-02	2.46E-01	2.34E-01	2.75E-01	2.41E-01
IMP	1.34E-01	2.32E-02	5.71E-02	2.02E-02	5.13E-02
ATP	1.38E-03	8.18E-04	2.07E-02	3.08E-02	3.28E-03
ADPR	5.92E-01	1.39E-02	4.10E-03	1.14E-02	1.37E-02

NC ctl-M vs MPA-M (for Fig. 9B)

**CZ017**	**TTEST (p-value) fraction**				
**Isotopologues**	**C0D0**	**DCx**	**C*Dx**	**Total***	
1,3BPG	9.92E-01	9.98E-01		9.92E-01	
S1,7BP	1.03E-01	2.61E-01		1.03E-01	
F1,6BP	4.47E-02	4.38E-02	7.60E-02	4.47E-02	
R1P	1.67E-01	1.67E-01		1.67E-01	
PRPP	1.40E-03	1.27E-03		1.40E-03	
**Isotopologues**	**C0N0D0**	**DCxNx**	**N*CxDx**	**C*NxDx**	**Total***
GSH	7.44E-01	1.48E-01		6.80E-01	7.44E-01
IMP	2.92E-01	9.27E-01	9.35E-02	1.45E-01	2.92E-01
ATP	1.13E-02	7.68E-04	1.40E-02	2.01E-01	1.13E-02
ADPR	4.02E-01	1.68E-01	3.68E-03	1.29E-01	4.02E-01

CA ctl-M vs MPA-M (for Fig. 9B)

**CZ017**	**TTEST (p-value) fraction**				
**Isotopologues**	**C0D0**	**DCx**	**C*Dx**	**Total***	
1,3BPG	4.75E-02	5.10E-02		4.75E-02	
S1,7BP	4.26E-03	2.83E-01		4.26E-03	
F1,6BP	4.05E-03	4.08E-03	7.19E-02	4.05E-03	
PRPP	3.74E-02	3.48E-02		3.74E-02	
R1P	4.37E-03	4.17E-03		4.37E-03	

Isotopologues	C0N0D0	DCxNx	N*CxDx	C*NxDx	Total*
GSH	2.64E-01	2.41E-01		1.90E-01	2.64E-01
Inosine	3.95E-03	1.47E-01	2.70E-01	4.73E-02	3.95E-03
IMP	6.92E-01	5.99E-01	3.26E-01	9.22E-01	6.92E-01
ATP	8.87E-01	2.83E-01	9.83E-01	7.60E-01	8.87E-01
ADPR	4.77E-01	4.83E-01	7.03E-01	8.50E-01	4.77E-01

NC ctl-T vs MPA-T T: main tissue + outgrowth (for Fig. 9B)

CZ017	TTEST (p-value) fraction				
**Isotopologues**	**C0D0**	**DCx**	**C*Dx**	**Total***	
1,3BPG	4.55E-01	4.49E-01		4.55E-01	
S1,7BP	2.60E-01	1.66E-01		2.60E-01	
F1,6BP	8.70E-02	8.79E-02	6.39E-03	8.70E-02	
PRPP	1.83E-03	8.48E-02		1.83E-03	
R1P	6.25E-02	5.96E-02		6.25E-02	
**Isotopologues**	**C0N0D0**	**DCxNx**	**N*CxDx**	**C*NxDx**	**Total***
GSH	7.03E-01	8.48E-02		1.96E-01	3.29E-01
Inosine	2.98E-03	1.31E-01	1.16E-02	2.99E-03	2.98E-03
IMP	8.92E-01	2.71E-01	2.23E-01	4.10E-01	8.92E-01
ATP	1.15E-01	1.33E-01	4.19E-03	2.66E-01	1.15E-01
ADPR	3.41E-01	3.85E-01	3.03E-02	2.55E-02	3.41E-01

CA ctl-T vs MPA-T T: main tissue + outgrowth (for Fig. 9B)

CZ017	TTEST (p-value) fraction				
**Isotopologues**	**C0D0**	**DCx**	**C*Dx**	**Total***	
1,3BPG	4.07E-01	4.96E-01		4.07E-01	
S1,7BP	4.18E-01	4.54E-01		4.18E-01	
F1,6BP	5.88E-02	5.85E-02	3.63E-01	5.88E-02	
PRPP	5.37E-02	1.15E-01		5.37E-02	
R1P	3.65E-01	3.89E-01		3.65E-01	
**Isotopologues**	**C0N0D0**	**DCxNx**	**N*CxDx**	**C*NxDx**	**Total***
GSH	1.84E-01	2.00E-01		1.77E-01	1.84E-01
Inosine	1.36E-02	7.29E-03	4.19E-01	1.37E-01	1.36E-02
IMP	4.44E-01	9.69E-02	7.45E-01	2.15E-01	4.44E-01
ATP	4.58E-01	2.74E-01	6.35E-01	9.09E-01	4.58E-01
ADPR	8.04E-01	9.63E-01	5.27E-01	1.86E-01	8.04E-01

T-tests for RPPA analysis (for Fig. 9C)

CZ017	TTEST (p-value) Normalized intensity			
	**NC-M**	**CA-M**	**NC-T**	**CA-T**
**Protein target**	**Ctl vs MPA**	**Ctl vs MPA**	**Ctl vs MPA**	**Ctl vs MPA**

PFKP	1.40E-02	1.68E-02	4.50E-02	2.17E-01
LDHA	9.26E-03	6.18E-02	7.62E-02	7.98E-02
PCK2	1.08E-02	1.03E-01	1.96E-02	5.12E-01
FBP1	6.84E-03	7.28E-03	4.76E-01	2.08E-01
PGD	2.11E-02	6.87E-04	7.24E-02	2.14E-01
ALDOA	4.08E-01	6.97E-01	1.72E-01	5.72E-01
SLC1A5	9.12E-02	3.82E-02	2.50E-02	1.41E-02
GLS	1.27E-01	1.12E-02	2.68E-01	3.11E-01
GLS2	9.07E-02	6.11E-02	9.83E-02	1.09E-01
GOT2	5.97E-03	2.61E-01	1.55E-03	8.53E-02
PPAT	6.13E-04	9.09E-03	6.99E-03	2.05E-01
PNP	9.68E-03	1.99E-01	5.21E-03	2.50E-01
